# Real‐Life Safety of Japanese Cedar Pollen Sublingual Immunotherapy Tablets: A Post‐Marketing Survey

**DOI:** 10.1002/clt2.70157

**Published:** 2026-02-13

**Authors:** Minoru Gotoh, Yuriko Maekawa, Tsuyoshi Ando, Noboru Kato, Eiji Horikawa, Noriaki Nishino

**Affiliations:** ^1^ Department of Otorhinolaryngology Nippon Medical School Tokyo Japan; ^2^ Department of Medical Information Pharmacovigilance & Quality Assurance Group Torii Pharmaceutical Co. Ltd. Tokyo Japan; ^3^ Department of Pharmacovigilance Pharmacovigilance & Quality Assurance Group Torii Pharmaceutical Co. Ltd. Tokyo Japan

**Keywords:** allergic rhinitis, Japanese cedar pollen, post‐marketing surveillance, SLIT, sublingual immunotherapy tablets

## Abstract

**Background:**

Japanese cedar (JC) pollen sublingual immunotherapy (SLIT)‐tablets (5000 Japanese allergy units [JAU]) are licensed for the treatment of JC‐pollinosis with no age restriction on the basis of the results of a 5‐year clinical trial. However, there have been no large‐scale surveys of 5000 JAU in an actual clinical setting.

**Methods:**

This was a multicenter observational prospective study. We assessed the safety and effectiveness of the long‐term use of 5000 JAU in patients with JC‐pollinosis, with an observation period of two seasons of JC pollen dispersal, at clinical sites in Japan.

**Results:**

Our safety analysis included 516 patients and the effectiveness analysis included 469 patients. Adverse drug reactions (ADRs) occurred in 68 patients (13.18%) and mainly comprised administration site‐related events that occurred during the early phase of administration. Treatment discontinuation due to ADRs occurred in 18 patients (3.49%). No deaths, anaphylactic shock, or serious ADRs occurred. Regarding effectiveness, the severity of JC‐pollinosis was rated as “almost asymptomatic + mild” in 82.19% of patients in Season 1 and 92.58% in Season 2. Quality of life was rated as “score 0 (Fine) + 1” in 75.83% of patients in Season 1 and 86.09% in Season 2. Overall improvement was rated as “improved + slightly improved” in 95.68% of patients in Season 1 and 96.38% in Season 2 following the initiation of JC pollen SLIT‐tablets. Nasal and ocular symptom scores also decreased with increasing treatment duration. Treatment continuation rates were 89.53% in Season 1 and 78.29% in Season 2.

**Conclusion:**

The JC pollen SLIT‐tablets appear to be safe and effective in an actual clinical setting during two seasons. No new safety or effectiveness issues were identified, and no additional safety or effectiveness precautions were required.

## Graphical abstract

We assessed the incidence of adverse drug reactions and effectiveness through two seasons of daily Japanese cedar (JC) pollen SLIT‐tablets 5000 JAU in Japan. Real‐life safety and effectiveness of JC pollen SLIT‐tablets appeared in patients with JC‐pollinosis.

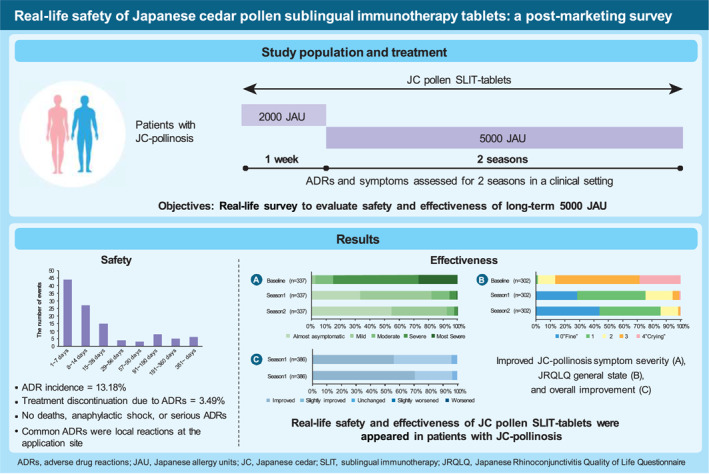

## 1 Introduction

In Japan, allergic rhinitis (AR) is a common allergic disease [1, 2, 3]. Seasonal AR and perennial AR are increasingly prevalent [1]; in particular, Japanese cedar (JC)‐pollinosis increased in prevalence from 16.2% in 1998 to 26.5% in 2008 and 38.8% in 2019, and prevalence has also increased in children (the prevalence among 5‐ to 9‐year olds was 30.1% in 2019) [2, 3]. The number of severe cases is also increasing. Currently in Japan, pollen allergen avoidance, symptomatic treatment, and allergen immunotherapy (AIT) are promoted as individual countermeasures against JC‐pollinosis [1, 3]. Therapy (i.e., the choice and combination of symptomatic medications) for pollinosis is selected based on disease severity (mild, moderate, or severe/most severe) and disease type (sneezing and rhinorrhea type, nasal blockade type, or combined type with nasal blockade as the chief complaint) in Japan [3]. Unlike typical symptomatic drug therapy, AIT aims to modify the natural course of allergic diseases via administration of the causative allergen [4], and treatment guidelines recommend AIT for all cases of “initial therapy” for “mild,” “moderate,” and “severe” AR [3]. AIT decreases symptoms and medication use, and improves patient quality of life [5, 6, 7, 8, 9, 10, 11, 12, 13, 14]. These benefits have been shown to persist after treatment if AIT is administered for 3–5 years [5, 6, 7, 8, 9, 10, 11, 12, 13, 14].

JC pollen sublingual immunotherapy (SLIT)‐tablets (Cedarcure, 2000 and 5000 Japanese Allergy Unit [JAU], Torii, Tokyo, Japan), fast‐dissolving lyophilized sublingual tablets, were approved in 2017 for the treatment of JC‐pollinosis in adult and pediatric patients without age restriction. In 2018, our house dust mite (HDM) fast‐dissolving SLIT‐tablets were also approved for the additional indication of HDM allergic rhinitis in pediatric patients under 12 years of age (without age restriction). Currently in Japan, JC pollen or HDM SLIT‐tablets can be prescribed to patients of all ages for whom sublingual administration is deemed feasible. In particular, this has facilitated the accumulation of clinical use cases for patients younger than 12 years old.

JC pollen SLIT‐tablets showed sustained clinical efficacy during 3 years of treatment and continued disease‐modifying effects for at least 2 years after treatment in a 5‐year clinical trial [14, 15, 16]. However, compared with actual clinical practice, the clinical trial setting features patients with a limited variety of backgrounds.

A recently reported real‐world compliance study of SLIT‐tablets was conducted using prescribing data from 2015 to 2021 in Japan. It reported that more patients maintained higher compliance with JC pollen SLIT‐tablets than with HDM SLIT‐tablets [17]. JC pollen SLIT‐tablets were also administered more frequently in younger patients (35.5% < 10 years and 40.5% 10–19 years), and among the recipients, 24.1% had asthma and 47.0% had atopic dermatitis/eczema. The package insert prohibits initiating administration of this drug during the JC pollen dispersal period, so administration was primarily initiated between May and November in actual clinical practice [17].

We report the results of a post‐marketing special drug‐use survey conducted as a requirement of the Pharmaceuticals and Medical Devices Agency (PMDA) upon approval of the JC pollen SLIT‐tablets in 2017. To evaluate the long‐term safety and effectiveness of daily JC pollen SLIT‐tablets 5000 JAU in Japan, we assessed the incidence of adverse drug reactions (ADRs) and effectiveness through two seasons of 5000 JAU administration. In addition, based on actual use, stratified analyses were conducted as post hoc analyses regarding age, comorbidities, JC pollen sensitization status, and JC‐pollinosis severity.

## 2 Methods

Details of this post‐marketing surveillance study are available in Supporting Information S1.

### 2.1 Study Population

This survey was conducted among patients with JC‐pollinosis, with an observation period of two allergen dispersal seasons, to investigate the safety and effectiveness of 5000 JAU under actual conditions of use. Patients were administered a 5000 JAU tablet daily, after taking a 2000 JAU tablet daily for 1 week.

### 2.2 Institutional Review Board Approval

This survey was approved by the Institutional Review Board of each participating site, and complied with all relevant regulations of the PMDA. We conducted the survey in accordance with the International Conference on Harmonization Pharmacovigilance Plan E2E guidelines [18] and Good Post‐marketing Study Practice [19]. It also complied with Good Pharmacovigilance Practices principles [20]. The patient provided informed consent.

### 2.3 Study Design

The JC pollen SLIT‐tablets (Cedarcure, Torii, Tokyo, Japan; manufactured by ALK‐Abelló, Hørsholm, Denmark) are fast‐dissolving freeze‐dried tablets of 2000 JAU (up‐dosing dose) or 5000 JAU (maintenance dose). The Japanese common unit for JC pollen SLIT products is the JAU. The Japanese Society of Allergology Task Force defined 12.5 mg/mL of Cry j 1, the major allergen of JC pollen, as 10,000 JAU/mL in 1996 [21]. JC pollen SLIT‐tablets with nominal strengths of 2000 and 5000 JAU have a Cry j 1 content corresponding to the stated potency as calculated with 10,000 JAU/mL JC pollen standard extracts.

This multicenter observational study (jRCT1080224132) prospectively enrolled patients from 103 clinical sites throughout Japan from October 2018 to December 2020. Each medical facility used a commercially available web‐based electronic data collection system (PostMaNet; Fujitsu FIP Ltd.) to collect and manage data. Data were collected by electronic case report forms (CRFs) at the start of treatment (baseline) and in Seasons 1 and 2. Symptoms at baseline were assessed within 3 years prior to drug initiation. In patients of discontinued administration, that timepoint was the final evaluation time.

Survey items were as follows: patient background, diseases covered in this survey, medical history/comorbidities, allergies other than JC pollen, 5000 JAU administration status, use of other drugs, allergen immunotherapy status, implementation of other therapies, severity of JC‐pollinosis symptoms, general quality of life status according to the Japanese Rhinoconjunctivitis Quality of Life Questionnaire (JRQLQ) [3, 22, 23, 24], overall improvement, JC pollen‐specific immunoglobulin E (IgE) antibody test, adverse events, and laboratory abnormalities related to adverse events.

### 2.4 Objectives

The evaluation of the primary safety objective was conducted based on ADR (defined as incidence of study drug‐related adverse events) and serious ADR frequency during the survey period, and ADR incidence analyzed by patient background factor. We coded ADRs with preferred terms using MedDRA/J version 26.0. Shock and anaphylaxis, including anaphylaxis‐related symptoms, were designated as important investigation items in the Japanese risk management plan.

The evaluation of primary effectiveness considered JC‐pollinosis symptom severity, JRQLQ general state, and overall improvement during the JC pollen dispersal period (January 1 to April 30 in Seasons 1 and 2) throughout the survey period. These items were assessed by physicians at patient visits between May 1 and 31 of each season (evaluation timepoint), based on symptoms during the JC pollen dispersal period (January 1 to April 30), and recorded in the CRFs. The factorial analysis included the improvement rate (percentage of patients who showed “at least one level of improvement”) of JC‐pollinosis symptom severity in Seasons 1 and 2, and patient background factors. As a post hoc analysis, cumulative incidences were calculated for “almost asymptomatic” and “at least one level of improvement” in terms of JC‐pollinosis symptom severity. The stratified analysis of JRQLQ general state scores included age (≤ 11 years, 12–17 years, and ≥ 18 years), comorbidities, and JC pollen‐specific IgE class (< 17.5 UA/mL, ≥ 17.5 UA/mL).

### 2.5 Statistical Analysis

Details of the statistical analyses are shown in Supporting Information S1.

To examine effectiveness under actual usage conditions, regardless of annual variation in JC pollen dispersal (large, medium, or small), we utilized efficacy rates for JRQLQ general state from published data on JC pollen SLIT drops and JC pollen SLIT‐tablets. We estimated treatment continuation rates for this drug to be 80% in the first year and 50% in the second year. Furthermore, the number of patients who fulfilled the conditions necessary to secure a safety analysis set was calculated, and the patient population required for the survey over two JC pollen dispersal seasons was estimated to be 500 patients.

Case acceptance or rejection was determined by the case review board.

The safety analysis set excluded from the patient population those patients who met at least one of the following conditions: (1) violation of contract (patient registration was outside the contract period or the start date of administration of the study drug was not within the contract period), (2) not administered (patients not receiving the study drug), (3) duplicate violation (patients with the same patient background [i.e., patient initials, date of birth, and sex] on the questionnaire), and (4) patients deemed inappropriate for safety evaluation.

The effectiveness analysis set further excluded patients from the safety analysis set who met at least one of the following conditions: (5) registration violation (patients not registered within 30 days of the start date of drug administration); (6) patients with a history of administration of this drug; (7) patients with a history of administration of JC pollen SLIT drops; (8) at all observational points, patients with “Unknown” or “Unable to judge” recorded for overall improvement, and patients with missing entries for JC‐pollinosis symptom severity, symptom score, and JRQLQ general state; (9) patients who provided consent after the enrollment date; (10) patients who were not administered this drug during any of the following JC pollen dispersal seasons, including those who discontinued use before the JC pollen dispersal period (∼December 31) during Season 1, and those who never received the drug during the JC pollen dispersal period of Season 1 (January 1 to April 30 of the year following the start of treatment) or Season 2 (January 1 to April 30 two years after the start of treatment); and (11) patients deemed inappropriate for effectiveness evaluation.

For the safety analysis, adverse events were classified by system organ class, and the incidence rate was examined by preferred term. In addition, to examine the investigation items and the frequency of ADRs, we calculated the incidence rate and performed the *χ* ^2^ test or Fisher's direct probability calculation.

For the effectiveness analysis, a paired *t*‐test or Wilcoxon signed‐rank test was performed on the JRQLQ general state scores before and after administration to examine the change in effectiveness score from baseline. The Kaplan–Meier method was used to calculate the cumulative incidence of improvement in JC‐pollinosis symptom severity. For patients who experienced the “event of interest,” the number of days from the date of first administration to the first time point at which either of the following events of interest occurred was analyzed: (1) cedar pollinosis symptom severity rating after administration became “almost asymptomatic,” or (2) the severity rating after administration improved by “at least one level of improvement” compared with baseline. For censored patients who did not have the event of interest, the number of days from the date of the first dose to the date of the last observation was used. In either analysis pattern, patients who were “almost asymptomatic” at baseline were excluded. Furthermore, a log‐rank test was performed to stratify the data by JC‐pollinosis symptom severity at baseline according to the categories “age,” “mono‐ or poly‐sensitization,” and “single or dual SLIT (with or without HDM SLIT‐tablets).”

Regardless of whether the survey items were continuous or discrete, the required summary statistics were expressed as mean ± standard deviation, number of patients (n), and frequency (%). In all tests, *p*‐values less than 0.05 were treated as significant.

All statistical tests were performed with the statistical analysis system (SAS) software version 9.3 or higher (SAS Institute, Cary, NC, US).

## 3 Results

### 3.1 Patient Disposition

**FIGURE 1:**
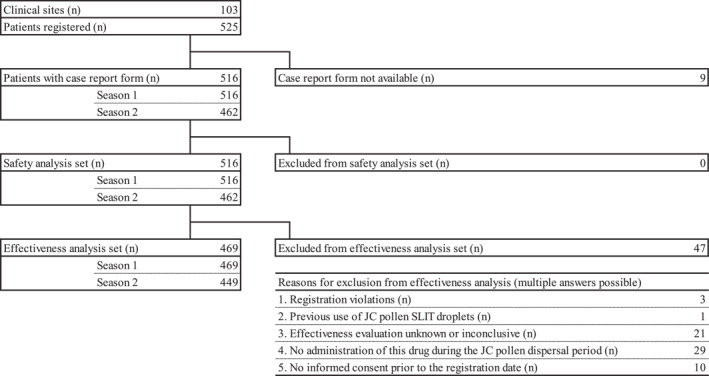
Patient disposition.

As of March 31, 2023, 525 patients were enrolled from 103 sites, and 516 CRFs were collected (Figure 1).

The safety analysis set included the 516 patients for whom a CRF was collected. The effectiveness analysis set included 469 patients, excluding from the safety analysis set 47 on the basis of “registration violations,” “previous use of JC pollen SLIT droplets,” “effectiveness evaluation unknown or inconclusive,” “no administration of this drug during the JC pollen dispersion,” and “no informed consent prior to the registration date.”

### 3.2 Patient Characteristics at Baseline

Table 1 shows the patient background data and treatment status in the safety analysis (*n* = 516) and effectiveness analysis (*n* = 469). The safety analysis included 279 (54.07%) male patients. The mean (± standard deviation) age at baseline was 23.2 (± 17.7) years, and 42.25% were ≤ 11 years, 16.47% were 12–17 years, and 41.28% were ≥ 18 years. The mean duration of JC‐pollinosis was 7.8 years. JC‐pollinosis symptom severity at baseline was most commonly “severe” (241 patients, 46.71%), followed by “most severe” (128 patients, 24.81%). At baseline, 83.91% of patients had JC‐pollen specific IgE levels of ≥ 3.5 UA/mL. Comorbidities were recorded in 59.30% (*n* = 306) of patients, of whom 58 had asthma and 43 had atopic dermatitis. Non–Japanese cedar (*Cryptomeria japonica*)‐pollen allergies were noted in 87.60% (*n* = 452) of patients, and the most common allergen was Japanese cypress (*Chamaecyparis obtusa*) pollen (*n* = 330, 73.01%), followed by HDM (*n* = 289, 63.94%).

**TABLE 1:** Patient demographics and characteristics at baseline.

Variables			Safety analysis set		Effectiveness analysis set	
			Patients, *n*	%	Patients, *n*	%
Analysis set			516		469	
Sex	Male		279	54.07	252	53.73
	Female		237	45.93	217	46.27
	Pregnancy	No	234	98.73	216	99.54
		Yes	3	1.27	1	0.46
Age		≤ 11 years	218	42.25	205	43.71
		12–17 years	85	16.47	75	15.99
		≥ 18 years	213	41.28	189	40.30
	Summary statistics	Patients, n	516		469	
		Mean ± standard deviation	23.2 ± 17.7		22.8 ± 17.5	
		Median	13.0		13.0	
		Minimum value–maximum value	3–82		3–82	
Comorbidities	No		202	39.15	190	40.51
	Yes		306	59.30	273	58.21
	Disease^a^	Asthma	58	18.95	54	19.78
		Atopic dermatitis	43	14.05	37	13.55
		Urticaria	4	1.31	4	1.47
		Allergic rhinitis	253	82.68	229	83.88
		Food allergy	21	6.86	20	7.33
		Hypertension	5	1.63	4	1.47
		Dyslipidemia	9	2.94	7	2.56
		Diabetes mellitus	3	0.98	3	1.10
		Sinusitis	7	2.29	7	2.56
		Others	52	16.99	44	16.12
		Allergic conjunctivitis	20	6.54	19	6.96
		Liver disease	0	0.00	0	0.00
		Kidney disease	1	0.33	0	0.00
	Unknown		8	1.55	6	1.28
Duration of JC‐pollinosis		< 1 year	25	4.84	24	5.12
		≥ 1 to < 3 years	55	10.66	50	10.66
		≥ 3 to < 6 years	67	12.98	66	14.07
		≥ 6 to < 11 years	65	12.60	59	12.58
		≥ 11 to < 16 years	14	2.71	14	2.99
		≥ 16 to < 21 years	12	2.33	11	2.35
		≥ 21 years	27	5.23	26	5.54
		Unknown	251	48.64	219	46.70
	Summary statistics	Patients, n	265		250	
		Mean ± standard deviation	7.8 ± 8.9		7.9 ± 9.1	
		Median	5.0		4.5	
		Minimum value–maximum value	0–54		0–54	
JC pollen‐specific IgE (baseline)		< 0.35 UA/mL	0	0.00	0	0.00
		≥ 0.35 to < 0.7 UA/mL	6	1.16	5	1.07
		≥ 0.7 to < 3.5 UA/mL	38	7.36	33	7.04
		≥ 3.5 to < 17.5 UA/mL	124	24.03	111	23.67
		≥ 17.5 to < 50 UA/mL	118	22.87	110	23.45
		≥ 50 to < 100 UA/mL	91	17.64	83	17.70
		≥ 100	100	19.38	91	19.40
		Not measured	39	7.56	36	7.68
JC‐pollinosis symptom severity (baseline)		Most severe	128	24.81	117	24.95
		Severe	241	46.71	233	49.68
		Moderate	60	11.63	56	11.94
		Mild	12	2.33	10	2.13
		Almost asymptomatic	1	0.19	1	0.21
		Unknown	74	14.34	52	11.09
Previous treatment for JC‐pollinosis		No	132	25.58	122	26.01
		Yes	305	59.11	282	60.13
		Unknown	79	15.31	65	13.86
Concomitant drugs^b^		No	150	29.07	129	27.51
		Yes	366	70.93	340	72.49
Treatment duration		≤ 7 days	5	0.97	0	0.00
		> 7 to ≤ 14 days	1	0.19	0	0.00
		> 14 to ≤ 28 days	2	0.39	0	0.00
		> 28 to ≤ 180 days	33	6.40	11	2.35
		> 180 to ≤ 270 days	16	3.10	12	2.56
		> 270 to ≤ 360 days	20	3.88	19	4.05
		> 360 to ≤ 540 days	154	29.84	149	31.77
		> 540 to ≤ 630 days	90	17.44	88	18.76
		> 630 to ≤ 720 days	173	33.53	168	35.82
		> 720 days	22	4.26	22	4.69
		Unknown	0	0.00	0	0.00
Allergen immunotherapy (baseline)		No	464	89.92	422	89.98
		Yes	52	10.08	47	10.02
Allergen immunotherapy^c^ (after administration)		No	416	80.62	377	80.38
		Yes	100	19.38	92	19.62
HDM SLIT‐tablets as concomitant allergen immunotherapy		No	414	80.23	375	79.96
		Yes	102	19.77	94	20.04
Allergies other than JC pollen allergens (baseline)	No		54	10.47	49	10.45
	Yes		452	87.60	410	87.42
	Allergens^d^	Mite	289	63.94	259	63.17
		House dust	237	52.43	214	52.20
		Dog	69	15.27	65	15.85
		Cat	94	20.80	88	21.46
		Moth	36	7.96	34	8.29
		Cypress pollen	330	73.01	302	73.66
		Orchard grass pollen	139	30.75	129	31.46
		Ragweed pollen	102	22.57	94	22.93
		Mugwort pollen	52	11.50	48	11.71
		Alder pollen	78	17.26	72	17.56
		Timothy grass pollen	41	9.07	39	9.51
		Sweet vernal grass pollen	9	1.99	9	2.20
		White birch pollen	48	10.62	46	11.22
		Egg white	7	1.55	7	1.71
		Milk	8	1.77	8	1.95
		Wheat	16	3.54	15	3.66
		Peanut	19	4.20	17	4.15
		Buckwheat	13	2.88	12	2.93
		Shrimp	7	1.55	7	1.71
		Crab	8	1.77	7	1.71
		Apple	16	3.54	14	3.41
		Kiwi	15	3.32	13	3.17
		Peach	4	0.88	3	0.73
		Celery	0	0.00	0	0.00
		Tomato	5	1.11	3	0.73
		Others	62	13.72	55	13.41
	Unknown		10	1.94	10	2.13

Abbreviations: HDM, house dust mite; IgE, immunoglobulin E; JC, Japanese cedar; SLIT, sublingual immunotherapy.

^a^ Multiple answers possible.

^b^ Concomitant drugs for which the reason for use was “adverse event treatment” were excluded.

^c^ Aggregate data from both Seasons 1 and 2.

^d^ Multiple answers possible.

### 3.3 Treatment Discontinuation

Of the 516 patients in the safety analysis set, 112 patients discontinued 5000 JAU: 54 (10.47%) and 58 (12.55%) in Seasons 1 and 2, respectively. The most common reason for discontinuation/ending treatment was “changing clinic/no clinic visiting,” which accounted for 61.11% (*n* = 33) of discontinuations in Season 1 and remained high at 91.38% (*n* = 53) in Season 2. “Adverse events” decreased from 25.93% (*n* = 14) in Season 1 to 1.72% (*n* = 1) in Season 2. “Insufficient response” ranged from 1.85% (*n* = 1) to 3.45% (*n* = 2) in Season 1 to Season 2, and there were no discontinuations owing to “symptoms disappeared.”

Treatment continuation rates were 89.53% (*n* = 462/516) in Season 1 and 78.29% (*n* = 404/516) in Season 2, with 58 patients discontinuing after Season 1. There were five patients where treatment was continued with a reduced dose as necessary.

### 3.4 Safety

The ADR incidence is shown in Table S1, and the incidence by patient background is shown in Table S2.

**FIGURE 2:**
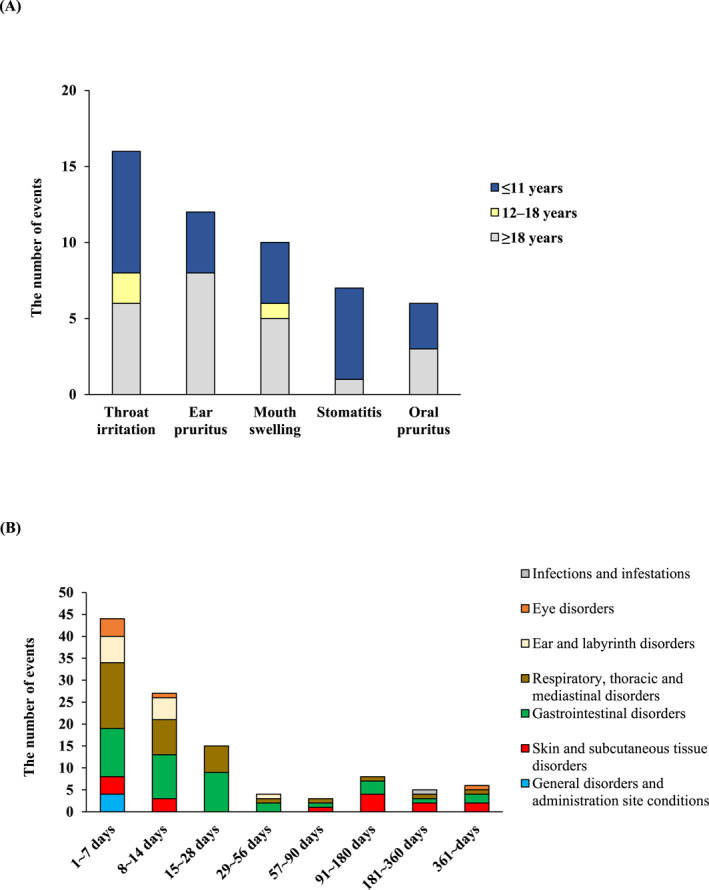
Most common ADRs (occurred in ≥ 1% of patients administered 5000 JAU) stratified by age (A) and onset of ADRs stratified by SOC classification (B) using the Japanese version of the International Council for Harmonization of Technical Requirements for Pharmaceuticals for Human Use Medical Dictionary for Regulatory Activities (MedDRA/J 26.0) in the safety analysis set (*n* = 516). ADR, adverse drug reaction; JAU, Japanese Allergy Units; SOC, system organ class.

In the safety analysis set, 112 ADRs occurred in 68 patients, with an incidence rate of 13.18% (Table S1). There were no deaths or instances of anaphylactic shock. The most common ADRs (≥ 1%) were throat irritation (2.91%), ear pruritus (2.33%), mouth swelling (1.94%), and stomatitis and oral pruritus (each 1.16%) (Figure 2A, Table S1). Only two occurred in ≥ 2% of patients. No serious ADRs were observed. There were no differences in the occurrences of these ADRs by age group.

Regarding the timing of occurrence, 44 ADR events (39.29%) occurred within 7 days after the start of administration, 27 events (24.11%) between 8 and 14 days, and 15 events (13.39%) between 15 and 28 days, for a total of 86 events (76.79%) within 28 days (Figure 2B). Regarding ADR outcomes, out of 112 events, 107 cases were deemed “recovered” or “relieved,” and 5 cases were categorized as “unknown.” ADRs led to discontinuation (including temporary treatment interruptions) in 32 events (18 patients, 3.49%) and dose reduction in 5 events (3 patients, 0.58%).

Regarding patient background factors and ADR incidence, significant differences were found in the following eight categories: “age group of elderly (< 65 years, ≥ 65 years),” “presence/absence of alcohol consumption,” “presence/absence of medical history,” “presence/absence of comorbidities,” “average daily dose (JAU),” “duration of treatment (days),” “presence/absence of previous treatment for JC‐pollinosis,” and “presence/absence of allergen immunotherapy (baseline).” However, no new items requiring attention were identified (Table S2, Supporting Information S1).

### 3.5 Effectiveness

**FIGURE 3:**
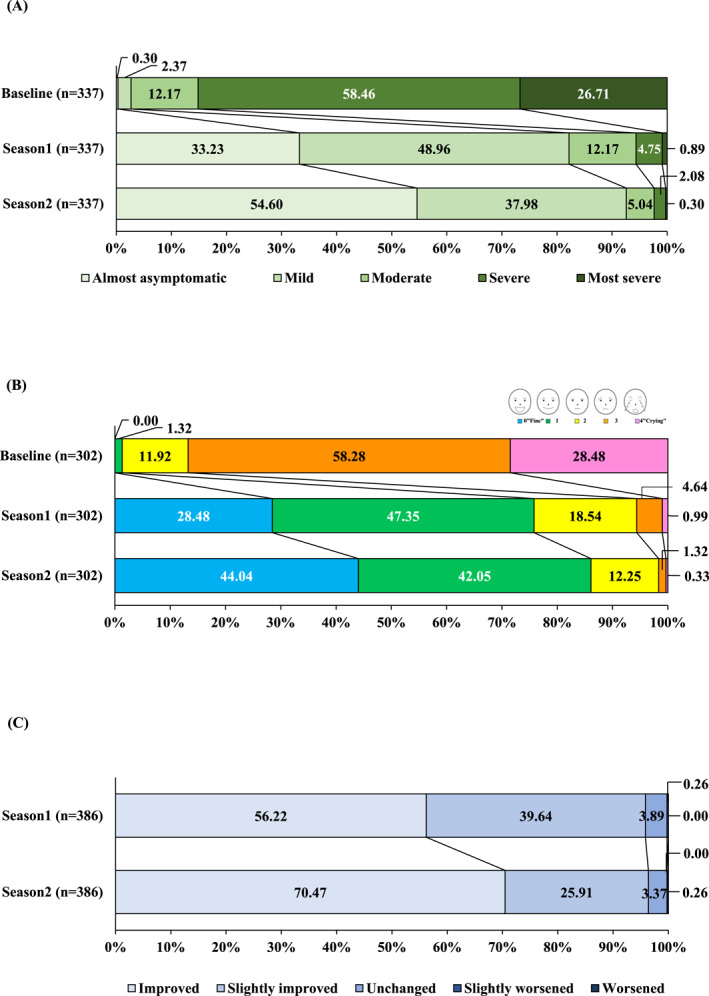
JC‐pollinosis symptom severity (A), JRQLQ general state (B), and overall improvement (C) over time in the effectiveness analysis set (*n* = 469). JC‐pollinosis symptom severity was based on a five‐point scale (data show the proportion of each scale). The JRQLQ general state was based on the face scale (data show the proportion of each score on the face scale). Overall improvement was based on a five‐point scale (data show the proportion of each score on the scale). A paired *t*‐test was used to assess the rhinitis symptom severity scores and JRQLQ general state at each timepoint corresponding to the baseline (*p* < 0.001 at all timepoints). JC, Japanese cedar; JRQLQ, Japanese Rhinoconjunctivitis Quality of Life Questionnaire.

In the effectiveness analysis, JC‐pollinosis symptom severity, JRQLQ general state, and overall improvement were evaluated in Seasons 1 and 2 (Figure 3).

In terms of severity, JC‐pollinosis symptoms were “most severe” in 26.71% of patients at baseline, decreasing to 0.89% in Season 1 and 0.30% in Season 2. The proportions of “almost asymptomatic + mild” were 82.19% in Season 1 and 92.58% in Season 2. The change in score distribution from baseline was statistically significant (*p* < 0.001) at all time points after Season 1. The incidence of each nasal and ocular symptom also decreased after Season 1 (*p* < 0.001 at all time points, Supporting Information S1: Figure S1). The mean changes in scores from baseline for total nasal symptoms were −5.7 for Season 1 and −6.6 for Season 2, for total ocular symptoms were −2.1 for Season 1 and −2.4 for Season 2, and for overall combined scores were −10.7 for Season 1 and −12.5 for Season 2.

Regarding the JRQLQ general state, the rate of score 4 (Crying) was 28.48% at baseline, but decreased to 0.99% in Season 1 and 0.33% in Season 2. The proportion of patients recorded as “score 0 (Fine) + 1” was 75.83% in Season 1 and 86.09% in Season 2. The change in score distribution from baseline reached a significance of *p* < 0.001 at all time points after Season 1.

Regarding overall improvement, the improvement rate (the percentage of patients recorded as “improved + slightly improved”) in Season 1 was 95.86% and 96.38% in Season 2.

**FIGURE 4:**
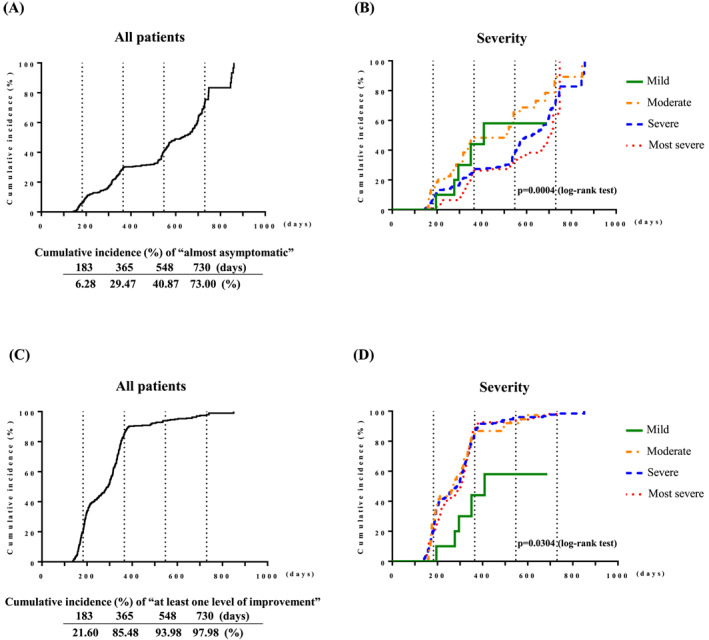
Cumulative incidence of “almost asymptomatic” (A, B) and “at least one level of improvement (C, D) status for JC‐pollinosis symptom severity after receiving JC pollen SLIT‐tablets. Regarding “almost asymptomatic,” (A) shows the results for all patients and (B) shows the stratified results by JC‐pollinosis symptom severity at baseline. Regarding “at least one level of improvement,” (C) shows the results for all patients and (D) shows the stratified results by JC‐pollinosis symptom severity at baseline. A log‐rank test was performed to stratify by severity of JC‐pollinosis symptoms at baseline in (B) and (D). JC, Japanese cedar; SLIT, sublingual immunotherapy.

The cumulative incidence of “almost asymptomatic” and “at least one level of improvement” in terms of JC‐pollinosis symptom severity is shown in Figure 4. After starting treatment, 6.28% of patients were “almost asymptomatic” at 0.5 years, 29.47% at 1 year, 40.87% at 1.5 years, and 73% at 2 years, but the percentage of patients who experienced “at least one level of improvement” was 21.6% at 0.5 years, 85.48% at 1 year, 93.98% at 1.5 years, and 97.98% at 2 years. In the stratified analysis, patients with milder disease severity at baseline were more likely to become “almost asymptomatic” (*p* = 0.0004, log‐rank) (Figure 4B), whereas “at least one level of improvement” was achieved faster in patients with moderate or higher severity (*p* = 0.0304, log‐rank) (Figure 4D). None of the results were affected by stratification by the categories “age” or “mono‐ or poly‐sensitization” (Supporting Information S1: Figure S3). In the stratification by “single or dual SLIT (with or without HDM SLIT‐tablets),” there was a significant difference in “at least one level of improvement” (*p* = 0.0465, log‐rank), but it was not deemed to have a substantive effect (Supporting Information S1: Figure S3).

The results of the stratified analysis of JRQLQ general state are shown in Supporting Information S1: Figure S2. In terms of age group, the proportion of “0 (Fine) + 1” scores increased over time in the groups ≤ 11 years, 12–17 years, and ≥ 18 years, with no differences by age. In the stratified analysis by category of comorbidity (no comorbidities, asthma, atopic dermatitis, and other comorbidities) and by JC pollen‐specific IgE class (< 17.5 UA/mL, ≥ 17.5 UA/mL), the proportion scored “0 (Fine) + 1” increased over time, and there were no clear differences by category. In the stratified analysis, the change in score distribution from baseline reached a significance of *p* < 0.001 at all time points after Season 1.

In the analysis of patient background factors that affected the improvement rate of JC‐pollinosis symptom severity, there were significant differences in six items. In Season 1, differences were observed in “presence/absence of comorbidities,” “presence/absence of allergen immunotherapy (baseline),” and “severity of JC‐pollinosis symptoms (baseline),” and in Season 2, differences were observed in “severity of JC‐pollinosis symptoms (baseline),” “age group (children, adults, elderly),” and “presence/absence of allergen immunotherapy (after administration of this drug).” However, no new items requiring attention were identified (Table S3, Supporting Information S1).

## 4 Discussion

The JC pollen SLIT‐tablets 5000 JAU have been tested in clinical trials for a total of 5 years (3 years of treatment and 2 years after treatment completion), and have demonstrated a favorable safety profile, efficacy depending on the duration of treatment, and disease‐modifying effects [14, 15, 16].

The World Allergy Organization indicates in its SLIT position paper that drug safety and effectiveness need to be assessed in the real world, where outcomes may differ from those of controlled clinical trials that provide premarket test results [25].

We report here the results of a real‐life observational survey conducted during a two‐season treatment period that included 516 patients prospectively enrolled from 103 sites in Japan. JC pollen dispersal varies greatly from year to year depending on climatic conditions. Previous studies in Japan have reported cumulative JC pollen dispersal ranging from 10,625 particles/cm^2^ in 2005 [26] to 1154 particles/cm^2^ in 2006 [27]. During the evaluation period of this survey (2019–2022), the cumulative JC pollen dispersal in central Tokyo was approximately 2500–5000 grains/cm^2^ (data not shown) [28], comparable to levels observed during the clinical trial period for this drug (2015–2019; 2894–5016 grains/cm^2^). In determining the sample size for this survey, calculations accounted for the potential influence of cumulative JC pollen dispersal. It is therefore considered unlikely that annual variation in natural pollen exposure had a significant impact on the evaluation of this drug in actual clinical practice. This survey showed that 5000 JAU administered for two seasons in a clinical setting showed favorable safety and effectiveness. In the safety evaluation, as in the existing clinical trial, the common ADRs were local reactions at the application site, and most of the ADRs were deemed “non‐serious.” These findings are similar to the safety profile observed in the existing clinical trial. The ADR incidence was 13.18%, which was lower than the incidence in the existing clinical trial (46.6%) [14]. This difference likely occurred because the present survey—a post‐marketing special drug‐use survey—was conducted under routine clinical practice in Japan, where information was obtained from medical records and physicians' comments, making it difficult to apply the active monitoring methods used in clinical trials. The high incidence observed in clinical trials is thought to result from proactive methods of investigation [29]. In addition, in this survey, the analysis of patient background factors did not find any association with a clear worsening of ADR incidence. No new cautions were required regarding the occurrence of ADRs by age.

The most common reason for the discontinuation of 5000 JAU was “changing clinic/no clinic visiting,” which exceeded 60% at all points. This frequency was similar to those found in the post‐marketing surveillance of other drugs in Japan (i.e., two HDM SLIT‐tablets and an HBV drug [30, 31, 32], and is believed to represent the actual clinical setting in Japan). “Adverse events” accounted for 25.93% of discontinuations in Season 1, but only 1.72% in Season 2. This high rate of discontinuation owing to ADRs in the first year was similar to the results reported earlier for the SQ‐HDM SLIT‐tablets after 24 months (25.15% at 6 months, 7.78% at 12 months, and 1.05%–2.33% at 24–36 months) [32]. These results indicate that in AIT, which must be continued for 3–5 years, the low dropout rate owing to ADRs is meaningful in an actual clinical setting, and that 5000 JAU contributes to AIT continuation from the viewpoint of safety.

In the effectiveness evaluation, recipients of 5000 JAU generally showed improvement from Season 1, with an increase or maintenance of improvement depending on the duration of administration. These findings were similar to those of the efficacy evaluation in the existing clinical trial. In addition, the decrease in the JRQLQ general state score after taking 5000 JAU was similar in the ≤ 11 years, 12–17 years, and ≥ 18 years age groups, and was not affected by the presence of comorbidities such as asthma and atopic dermatitis, nor by JC pollen sensitization status. These results provide meaningful information for real‐life 5000 JAU prescribing, especially for relatively young patients or in patients with asthma or atopic dermatitis.

In addition, in this survey, 21.6% of patients at 0.5 years after treatment initiation showed “at least one level of improvement” in JC‐pollinosis symptom severity, which was unaffected by the severity of JC‐pollinosis prior to administration. In the clinical trial, 5000 JAU significantly reduced the total nasal symptom and medication score during the peak symptom period by 32.1% (*p* < 0.001, 5000 JAU group in the first season) and 32.8% (*p* < 0.0001, Placebo‐to‐Active (5000 JAU) group, who took 5000 JAU for 3 months before the start of the second JC pollen dispersion season) compared with placebo, and this result has proven reproducible [14, 15]. As the evaluation items used in the clinical trial and this survey were different, a general comparison cannot be made; however, the “at least one level of improvement” observed in most patients from Season 1 in this survey may suggest that the efficacy of 3 months of administration observed in the clinical trial applies to actual clinical practice for patients with pollinosis of any severity. In actual clinical practice in Japan, 5000 JAU falls under the regulation stating that new dosing cannot be started during the JC pollen dispersal period, so this survey provides a meaningful outcome in the treatment environment that starts after JC pollen dispersal ends. In fact, medical receipt data confirmed that initial treatment with 5000 JAU was frequently performed between May and November [17]. Despite region‐specific variations in the timing of the JC pollen dispersal season, most patients experience allergic symptoms between mid‐February and late April [1]. Even if treatment begins around November, it would be possible to secure a treatment period of approximately 3 months prior to the next season, which is expected to be feasible and effective. Furthermore, 29.47% of patients at 1 year of treatment and 73% at 2 years of treatment were recorded as “almost asymptomatic” for JC‐pollinosis symptom severity, and the milder the severity before treatment, the faster they reached that designation. This is the first report to show that even the most severe patients of JC‐pollinosis can be rendered “almost asymptomatic” with the use of JC pollen SLIT‐tablets. The results of this survey support the policy outlined in the Japanese guidelines for the treatment of nasal allergies, which states that AIT should be introduced early, regardless of rhinitis severity.

Regarding the limitations of this survey, it was conducted as requested by the PMDA and was a single‐arm survey (meaning it lacked a comparator group). As such, we cannot discount the possibility of survival bias. There were missing data at each evaluation timepoint. Because this survey was not a clinical trial, detailed investigations of ADRs were not performed. The medication scores used to evaluate significant improvement in clinical trials of SLIT‐tablets for various allergens [13] were not included in the effectiveness evaluation because of difficulties in managing drug access and drug doses in an actual clinical setting, as well as the lack of a standardized medication scoring system in actual clinical practice in Japan. Therefore, the influence of increased access to, and dosage of, symptomatic medications on effectiveness cannot be completely ruled out. No new issues were identified with the effectiveness of the JC pollen SLIT‐tablets in this two‐season post‐marketing survey, which was conducted within the limits of lacking a control group and quantitative assessment of concomitant symptomatic medication. However, to address these limitations, further research using real‐world data that allow for longer‐term evaluation, such as electronic medical records or claims data, is needed. In addition, the sample size in some subgroups was insufficient to draw definitive conclusions. In this survey, the number of elderly patients enrolled was small; therefore, future studies in patients aged ≥ 60 years are warranted. Further research is also required to clarify the effects of SLIT on asthma and atopic dermatitis in patients with coexisting JC‐pollinosis.

## 5 Conclusion

In an actual clinical setting, the long‐term administration of 5000 JAU for two seasons was not likely to cause clinical problems or increase safety risks in patients with JC‐pollinosis. Regardless of JC‐pollinosis symptom severity, symptoms improved depending on the duration of administration; in some patients, symptoms were no longer detected. No additional safety or effectiveness measures were necessary.

## Author Contributions

**Minoru Gotoh:** conceptualization, writing – review and editing. **Yuriko Maekawa:** conceptualization, writing – original draft, writing – review and editing. **Tsuyoshi Ando:** data curation, formal analysis, visualization, writing – review and editing. **Noboru Kato:** conceptualization, writing – review and editing. **Eiji Horikawa:** investigation, writing – review and editing. **Noriaki Nishino:** supervision, writing – review and editing.

## Ethics Statement

This post‐marketing drug‐use survey was approved by the Institutional Review Board of each participating site, and complied with all relevant regulations of the Pharmaceuticals and Medical Devices Agency. We conducted the survey in accordance with the International Conference on Harmonization Pharmacovigilance Plan E2E guidelines and Good Post‐marketing Study Practice. It also complied with Good Pharmacovigilance Practices principles.

## Consent

The patient provided informed consent.

## Conflicts of Interest

M.G. has received honoraria from Torii, Taiho, Hisamitsu, Meiji Seika Pharma, and Novartis. Y.M., T.A., N.K., E.H., and N.N. are employees of Torii Pharmaceutical Co. Ltd.

## Supporting information


Supporting Information S1

**Figure S1:** Severity scores for nasal and ocular symptoms after receiving Japanese cedar pollen sublingual immunotherapy tablets.

**Figure S2:** Stratified analysis of the Japanese Rhinoconjunctivitis Quality of Life Questionnaire general state by age (≤ 11 years, 12–17 years, and ≥ 18 years) (A), comorbidities (B), and Japanese cedar pollen‐specific immunoglobulin E (< 17.5 UA/mL, ≥ 17.5 UA/mL) (C).

**Figure S3:** Stratified analysis of cumulative incidence of patients who were recorded as “almost asymptomatic” (A) and “at least one level of improvement” (B) for Japanese cedar pollinosis symptom severity after receiving Japanese cedar pollen sublingual immunotherapy tablets.

**Table S1:** Frequency and type of adverse drug reactions: Safety analysis set (*n* = 516).

**Table S2:** Frequency and type of adverse drug reactions by patient background (*n* = 516).

**Table S3:** Improvement rate (symptom severity of cedar pollen allergy) by patient background in effectiveness analysis set (*n* = 469).

## Data Availability

Research data are not shared.
