# Supplementary material for: Real‐Life Safety of Japanese Cedar Pollen Sublingual Immunotherapy Tablets: A Post‐Marketing Survey
Source: Clin Transl Allergy. 2026 Feb 13;16(2):e70157. doi: 10.1002/clt2.70157 (PMC12904777; doi:10.1002/clt2.70157)
Supplement: Supplementary file 1 — Supporting Information S1 [file CLT2-16-e70157-s001.docx]

Supporting Information

**Methods**

1. Study population

The participants were patients with Japanese cedar (JC)-pollinosis who had no experience using JC sublingual immunotherapy (SLIT)-tablets. The target number of patients was 500.

1. Survey method

The survey period was from August 27, 2018 to December 31, 2023, and the enrollment period was from October 1, 2018 to December 31, 2020. The observation period was two JC pollen dispersal seasons.

Patients were enrolled using a serial survey method, and were enrolled up to the 30th day after drug initiation. The survey was requested by the Pharmaceuticals and Medical Devices Agency and contracted to medical institutions that had open accounts of this drug. The case report form was divided into two booklets: Season 1 and Season 2 after the start of drug administration. If the administration of this drug was discontinued during the survey period, the observation period was defined as the period up to the discontinuation of the drug.

This survey was conducted in accordance with the Good Postmarketing Study Practice Ministerial Ordinance (Ministerial Ordinance on Standards for Conducting Post-marketing Surveillance and Testing of Pharmaceuticals: Ministry of Health, Labour and Welfare Ordinance No. 171, December 20, 2004).

1. Dosage and Administration

The usual dose is one Cedarcure JC pollen SLIT-tablet 2000 Japanese allergy unit (JAU) daily during the first week of treatment and one Cedarcure JC pollen SLIT-tablet 5000 JAU daily from the second week. Patients keep the tablet under the tongue for 1 minute and then swallow it. Patients are instructed to avoid gargling, eating, and drinking for 5 minutes after swallowing the tablet.

The first dose is administered under the supervision of a physician. The patient should rest and be monitored carefully for at least 30 minutes after the first dose. Preparations are made for acute care of patients with shock or anaphylaxis.

1. Survey item

We investigated patient information at the time of drug initiation, administration status, and the safety and effectiveness of the drug.

Patient information included the following: sex, date of birth, height, weight, inpatient/outpatient classification, lifestyle habits, family history, target disease of this survey, medical history/complications, presence or absence of JC pollen-specific immunoglobulin E antibody test, allergies other than JC pollen, use of other medicines, implementation of allergen immunotherapy, and implementation of other therapies.

Regarding the administration status of this drug, we investigated compliance status, start date of administration, end date of administration, interruption/discontinuation, and reasons for discontinuation.

Safety investigation items included adverse event term, date of onset, severity, administration of the drug, presence or absence of treatment, outcome, date of outcome, causal relationship with the drug, other suspected factors, and abnormal laboratory values related to the adverse event. Adverse drug reactions (ADRs) were defined as adverse events for which a causal relationship with this drug could not be ruled out.

Effectiveness investigation items included JC-pollinosis symptom severity, each nasal and ocular symptom, Japanese Rhinoconjunctivitis Quality of Life Questionnaire (JRQLQ) general state, and overall improvement from baseline after administration of this drug (Seasons 1 and 2). As a post hoc analysis, cumulative incidences were calculated for “almost asymptomatic” and “at least one level of improvement” in terms of JC-pollinosis symptom severity. The stratified analysis of JRQLQ general state scores included age (≤11 years, 12–17 years, and ≥18 years), comorbidities, and JC pollen-specific immunoglobulin E class (<17.5 UA/mL, ≥17.5 UA/mL).

1. Methods of tabulation and analysis

In this survey, we used an electronic data capture system, PostMaNet (Fujitsu FIP Ltd.), to register cases and collect data via the internet. The statistical analysis was outsourced to EPS Corporation.

We determined case acceptance or rejection by the case review board.

We confirmed that the survey and observation (administration) period for the group before exclusion from analysis was a summary statistic (n=516, median=552 days, max=845 days).

JC-pollinosis symptom severity was evaluated by the investigator using a five-point scale of “almost asymptomatic,” “mild,” “moderate,” “severe,” and “most severe,” based on JC-pollinosis condition at baseline and in Seasons 1 and 2. The ratio of each severity was calculated only for continuing patients at each evaluation timepoint.

The score for each nasal and ocular symptom was determined according to the 2016 edition of the Practical Guideline for the Management of Allergic Rhinitis in Japan (8th revised edition) for nasal discharge, nasal congestion, sneezing, and difficulty in daily life as follows: “4+” (most severe) = 4 points, “3+” (severe) = 3 points, “2+” (moderate) = 2 points, “1+” (mild) = 1 point, and “–” (asymptomatic) = 0 points. The average severity score for each symptom was calculated only for continuing patients at each evaluation timepoint.

The JRQLQ general state was determined using the scale described in “III. Overall status” in JRQLQ No. 1. It was evaluated using scores on a five-point scale, with Score 0 for “Fine” and Score 4 for “Crying,” and tabulations were made for patients for whom evaluations were obtained before the administration of the drug and at each evaluation timepoint.

Overall improvement was evaluated by the investigator using a five-point scale of “improved,” “slightly improved,” “unchanged,” “slightly worsened,” and “worsened,” based on the patient’s overall condition after 5000 JAU administration compared with that before 5000 JAU administration. Only patients continuing treatment at each evaluation timepoint were evaluated.

To examine the factors that influenced ADR incidence and JC-pollinosis symptom severity at the final evaluation, we conducted an analysis by patient background factor.

Regardless of whether the survey items were continuous or discrete, the required summary statistics were expressed as mean ± standard deviation, number of patients (n), and frequency (%). In all tests, p-values less than 0.05 were treated as significant.

All statistical tests were performed using SAS software version 9.3 or higher (SAS Institute, Cary, NC, US).

**Results**

1. Analysis of patient background factors affecting ADR incidence

Significant differences were found in the following eight categories: “age group of elderly (<65 years, ≥65 years),” “presence/absence of alcohol consumption,” “presence/absence of medical history,” “presence/absence of comorbidities,” “average daily dose (JAU),” “duration of treatment (days),” “presence/absence of previous treatment for JC-pollinosis,” and “presence/absence of allergen immunotherapy (baseline).”

1. Age group of elderly (<65 years, ≥65 years)

The ADR incidence by “age group of elderly” was 36.36% (4/11 patients) in patients aged ≥65 years, which was significantly higher than the incidence of 12.67% (64/505 patients) in patients aged <65 years (p=0.044).

All ADRs that occurred in patients aged ≥65 years were non-serious, and all six ADRs other than dysphonia and wheezing were also observed in patients <65 years. Dysphonia and wheezing occurred only in the “≥65 years (elderly)” patients, but both ADRs resolved without treatment.

1. Presence/absence of alcohol consumption

The ADR incidence by “presence of alcohol consumption” was 22.03% (13/59 patients), which was significantly higher than the incidence of 11.94% (48/402 patients) in patients categorized as “absence of alcohol consumption” (p=0.040). The ADR incidence in patients whose drinking status was “unknown” was 12.73% (7/55 patients).

Of the 12 events that occurred in patients who consume alcohol, 4 events—dysphonia, wheezing, oral mucosal blistering, and swollen tongue—occurred only in patients who consumed alcohol (the number of affected patients for each was 1 [1.69%]).

ADRs that showed ≥5% difference in incidence between patients with and without alcohol consumption were ear pruritus (absent: n=6, 1.49%; present: n=4, 6.78%) and mouth swelling (absent: n=6, 1.49%, present: n=4, 6.78%).

1. Presence/absence of medical history

The ADR incidence by “presence of medical history” was 25.58% (11/43 patients), which was significantly higher than the 12.04% (56/465 patients) of patients in the “absence of medical history” category (p=0.018). The ADR incidence in patients whose medical history status was “unknown” was 12.50% (1/8 patients).

Of the nine events that occurred in patients with a medical history, one event—lip pruritus—occurred only in patients with a medical history (n=1 [2.33%]). ADRs that showed ≥5% difference in incidence between patients with and without medical history were ear pruritus (absent: n=9, 1.94%; present: n=3, 6.98%) and mouth swelling (absent: n=7, 1.51%; present: n=3, 6.98%).

The medical history was classified according to the presence of “asthma, food allergy, urticaria, and others” to confirm ADR occurrence. The ADR incidence was 33.33% (6/18 patients) in patients with a history of “asthma,” 22.22% (2/9 patients) in patients with a history of “food allergies,” 40.00% (2/5 patients) in patients with a history of “urticaria,” and 16.67% (3/18 patients) in patients with a history of “other.” ADRs that occurred in multiple patients included oral swelling in two patients (11.11%) with a history of “asthma,” and throat irritation in two patients (11.11%) with a history of “other.”

1. Presence/absence of comorbidities

The ADR incidence by “presence of comorbidities” was 16.34% (50/306 patients), which was significantly higher than 8.42% (17/202 patients) in patients with an “absence of comorbidities” (p=0.018). The ADR incidence in patients whose comorbidities were “unknown” was 12.50% (1/8 patients).

Of the 30 events that occurred only in patients with comorbidities, the ADRs that occurred in multiple patients were stomatitis in six patients (1.96%), eye pruritus and rash in three patients each (0.98%), and asthma, oral discomfort, atopic dermatitis, and urticaria in two patients each (0.65%).

The comorbidities were classified as “allergic rhinitis, asthma, atopic dermatitis, food allergies, allergic conjunctivitis, and others” to confirm the occurrence of ADRs. The ADR incidence was 15.81% (40/253 patients) in patients with “allergic rhinitis,” 24.14% (14/58 patients) in patients with “asthma,” 16.28% (7/43 patients) in patients with “atopic dermatitis,” 23.81% (5/21 patients) in patients with “food allergy,” 15.00% (3/20 patients) in patients with “allergic conjunctivitis,” and 24.00% (12/50 patients) in “other” patients.

1. Average daily dose

The ADR incidence by “average daily dose” was 50.00% (4/8 patients) in patients with ≤2000 JAU, 83.33% (5/6 patients) in patients with >2000 JAU and ≤4,000 JAU, and 11.75% (59/502 patients) in patients with >4,000 JAU, with significant differences observed between the categories of average daily dose (p<0.001).

The numbers of patients with an “average daily dose” of ≤2000 JAU and >2000 JAU and ≤4,000 JAU (patients who did not reach the maintenance dose and received a lower dose) were small, and there was no consistent trend observed in ADR incidence by “average daily dose” category. Additionally, there were no ADRs reported in multiple patients for whom the “average daily dose” was “≤2000 JAU” or “>2000 JAU and ≤4000 JAU.”

1. Duration of treatment

The ADR incidence by treatment period was 40.00% (2/5 patients) for “within 7 days,” 100.00% (1/1 patient) for “>7 days but ≤14 days,” 0.00% (0/2 patients) for “>14 days but ≤28 days,” 30.30% (10/33 patients) for “>28 days but ≤180 days,” 12.50% (2/16 patients) for “>180 days but ≤270 days,” 15.00% (3/20 patients) for “>270 days but ≤360 days,” 12.34% (19/154 patients) for “>360 days but ≤540 days,” 8.89% (8/90 patients) for “>540 days but ≤630 days),” 10.40% (18/173 patients) for “>630 days but ≤720 days,” and 22.73% (5/22 patients) for “>720 days,” with a significant difference observed between the treatment period categories (p=0.006).

There was no consistent trend observed in ADR incidence by administration period. Furthermore, no consistent trend was observed in the incidence of side effects by type of ADR.

1. Presence/absence of previous treatment for JC-pollinosis

The ADR incidence in patients in the category “presence of previous treatment for JC-pollinosis” was 14.75% (45/305 patients), which was significantly higher than the rate of 6.82% (9/132 patients) for patients in the category “absence of previous treatment for JC-pollinosis” (p=0.026). The ADR incidence in patients whose comorbidities were categorized as “unknown” was 17.72% (14/79 patients).

Of the 23 events that occurred in patients who had previously been treated for JC-pollinosis, ADRs that occurred in multiple patients were ear pruritus in 10 patients (3.28%), oral pruritus in 4 patients (1.31%), pruritus in 3 patients (0.98%), and eye pruritus, oral discomfort, and atopic dermatitis in 2 patients each (0.66%).

The previous treatments for JC-pollinosis were classified by the category “other allergy medications (oral use), otolaryngological medications (topical use), ophthalmic medications (topical use) and others” to confirm the occurrence of ADRs. The ADR incidence was 14.14% (41/290 patients) for those categorized under “other allergy medications (oral use),” 10.00% (12/120 patients) for “otolaryngological medications (topical use),” 18.60% (16/86 patients) for “ophthalmic medications (topical use),” and 32.00% (8/25 patients) for “other.”

1. Presence/absence of allergen immunotherapy (baseline)

The ADR incidence for patients in the category “presence of allergen immunotherapy (baseline)” was 3.85% (2/52 patients), which was significantly lower than the rate of 14.22% (66/464 patients) for patients in the category “absence of allergen immunotherapy (baseline)” (p=0.031). Adverse reactions that occurred in patients receiving allergen immunotherapy (baseline) included throat tightness and pruritus in one patient each (1.92%).

1. Summary

Examination of the ADR incidence by patient background revealed significant differences for eight patient background factors, but no new items requiring attention were identified.

1. Analysis of patient background factors affecting the improvement rate (percentage of patients who showed “at least one level of improvement”) of JC-pollinosis symptom severity in Seasons 1 and 2

Significant differences were found in the improvement rates when patients were categorized by the following items in Season 1: “presence/absence of comorbidities,” “presence/absence of allergen immunotherapy (baseline),” and “severity of JC-pollinosis symptoms (baseline).” In Season 2, differences in improvement rates were observed in relation to the categories “severity of JC-pollinosis symptoms (baseline),” “age group (children, adults, elderly),” and “presence/absence of allergen immunotherapy (after administration of this drug).” However, no new items requiring attention were identified.

1. Presence/absence of comorbidities in Season 1

The improvement rate according to the presence or absence of comorbidities in Season 1 was significantly lower in patients with comorbidities (91.93%, 205/223 patients) than in patients without comorbidities (97.01%, 162/167 patients) (p=0.049).

However, in Season 2, the improvement rate in patients with comorbidities was 96.06% (195/203 patients) and in patients without, 97.84% (136/139 patients), with no significant difference (p=0.535).

We deemed the difference in improvement rates between those with and without comorbidities in Season 1 to have no clinical significance because no significant difference was observed in Season 2.

1. Presence/absence of allergen immunotherapy (baseline) in Season 1

The improvement rate in Season 1 for those with allergen immunotherapy was 81.82% (36/44 patients), which was significantly lower than the rate in patients without allergen immunotherapy (95.73%, 336/351 patients) (p=0.002).

In Season 2, the improvement rate in patients with allergen immunotherapy was 94.59% (35/37 patients) and in patients without allergen immunotherapy was 97.09% (300/309 patients), with no significant difference (p=0.333).

As there was no significant difference observed in Season 2, we considered the difference in improvement rate between those with and without allergen immunotherapy (baseline) in Season 1 to have no clinical significance.

1. JC-pollinosis symptom severity (baseline) in Seasons 1 and 2

Based on symptom severity, the improvement rate was 96.30% (104/108 patients) in patients with “most severe,” 96.85% (215/222 patients) in patients with “severe,” 90.74% (49/54 patients) in patients with “moderate,” 40.00% (4/10 patients) in patients with “mild,” and 0.00% (0/1 patient) in patients with “almost asymptomatic” JC-pollinosis.

In Season 2, the rate was 100.00% (95/95 patients) in patients with “most severe,” 97.51% (196/201 patients) in patients with “severe,” 97.56% (40/41 patients) in patients with “moderate,” 50.00% (4/8 patients) in patients with “mild,” and 0.00% (0/1 patient) in patients with “almost asymptomatic” JC-pollinosis.

A significant difference was observed in the improvement rates according to “severity of JC-pollinosis (baseline)” in Seasons 1 and 2 (both p<0.001).

We believe that the significant difference according to “severity of JC-pollinosis symptoms (baseline)” in Seasons 1 and 2 was related to the fact that patients with more severe JC-pollinosis symptoms at baseline were more likely to feel improvement.

1. Age group (children, adults, elderly) in Season 2

In Season 2, the improvement rate in patients categorized as children (<15 years) was 97.10% (201/207 patients), 97.04% (131/135 patients) in adults (≥15 to <65 years), and 75.00% (3/4 patients) in the elderly group (≥65 years). A significant difference was observed in the improvement rate according to the age group in Season 2 (p=0.044).

However, in Season 1, the improvement rates according to age group (children, adults, elderly) were 93.39% (212/227 patients) in children (<15 years), 95.65% (154/161 patients) in adults (≥15 to <65 years), and 85.71% (6/7 patients) in the elderly group (≥65 years), with no significant difference (p=0.405).

Although significant differences were observed by age in Season 2, this was thought to be caused by the small number of elderly patients (aged ≥65 years), and age is considered unlikely to affect effectiveness.

1. Presence/absence of allergen immunotherapy (after administration of this drug) in Season 2

In Season 2, the improvement rate in patients with allergen immunotherapy (after administration of this drug) was 92.31% (60/65 patients), which was significantly lower than the rate in patients without allergen immunotherapy (97.86%, 275/281 patients) (p=0.037).

In Season 1, the improvement rate in patients with allergen immunotherapy was 94.74% (18/19 patients) and that in patients without allergen immunotherapy was 94.15% (354/376 patients), which did not statistically differ (p=1.000).

In both Seasons 1 and 2, the improvement rate exceeded 90%, and it is unlikely that the effectiveness of this drug is affected by the presence or absence of allergen immunotherapy after administration.

We felt that the difference in improvement rates between those with and without allergen immunotherapy in Season 2 had no clinical significance because a significant difference was observed in Season 2, but not in Season 1.

1. Summary

As a result of examining the overall improvement at the final evaluation by patient background, significant differences were observed in six patient background factors, but no new items requiring attention were identified.

Figure legends

**Figure S1. Severity scores for nasal and ocular symptoms after receiving Japanese cedar pollen sublingual immunotherapy tablets**

The mean ± standard deviation of each nasal and ocular symptom severity score is shown. For each symptom, a paired t-test was used to assess the severity scores at each timepoint in relation to the baseline (for all items, p<0.001 at all timepoints).

**Figure S2. Stratified analysis of the Japanese Rhinoconjunctivitis Quality of Life Questionnaire general state by age (≤11 years, 12–17 years, and ≥18 years) (A), comorbidities (B), and Japanese cedar pollen-specific immunoglobulin E (<17.5 UA/mL, ≥17.5 UA/mL) (C).**

A paired t-test was performed on the Japanese Rhinoconjunctivitis Quality of Life Questionnaire general state score at each timepoint in relation to the baseline score (for all items, p<0.001 at all timepoints).

**Figure S3. Stratified analysis of cumulative incidence of patients who were recorded as “almost asymptomatic” (A) and “at least one level of improvement” (B) for Japanese cedar pollinosis symptom severity after receiving Japanese cedar pollen sublingual immunotherapy tablets.**

Stratified analyses were performed using the categories “age (≤11 years, 12–17 years, ≥18 years)”, “mono-sensitization or poly-sensitization,” and “single or dual SLIT (with or without HDM SLIT-tablets).” A log-rank test was performed for stratification by JC-pollinosis symptom severity at baseline in (A) and (B). HDM, house dust mite; JC, Japanese cedar; SLIT, sublingual immunotherapy.
