# Supplementary figures and images for: Real‐Life Safety of Japanese Cedar Pollen Sublingual Immunotherapy Tablets: A Post‐Marketing Survey
Source: Clin Transl Allergy. 2026 Feb 13;16(2):e70157. doi: 10.1002/clt2.70157 (PMC12904777; doi:10.1002/clt2.70157)

**Figure S1**

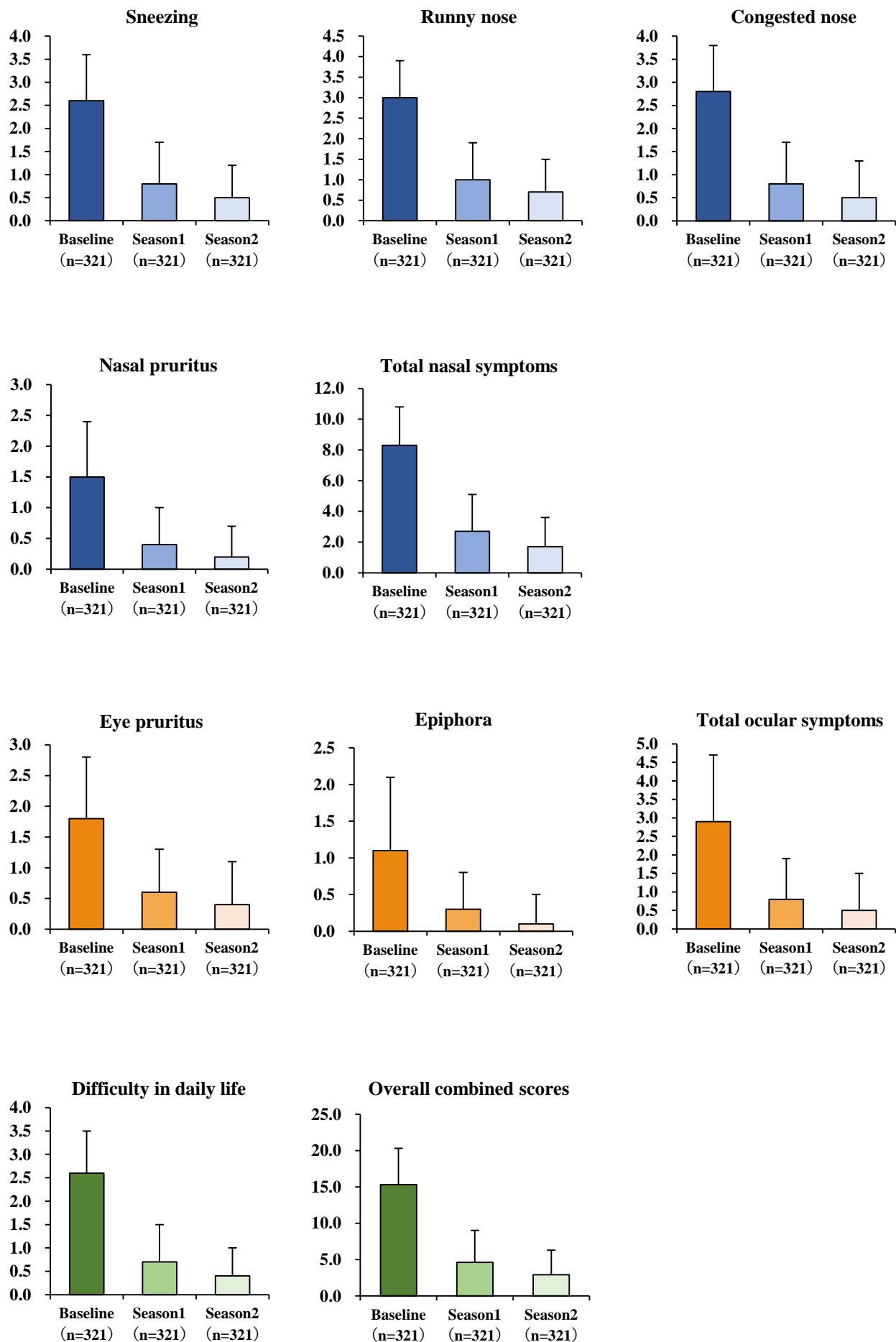

Supplement: Supplementary file 2 — Figure S1: Severity scores for nasal and ocular symptoms after receiving Japanese cedar pollen sublingual immunotherapy tablets. [file CLT2-16-e70157-s006.pdf]

Figure S2

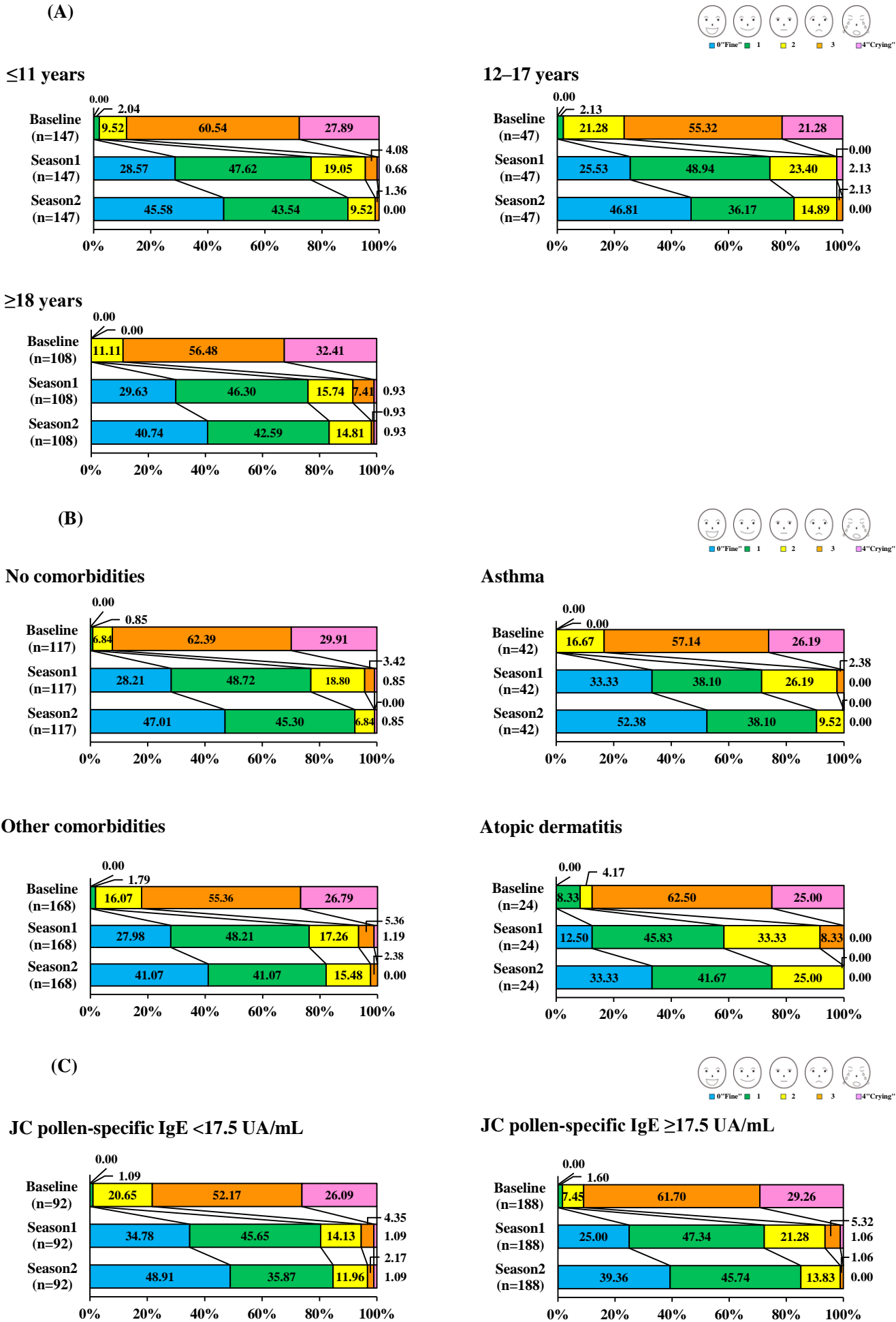

Supplement: Supplementary file 3 — Figure S2: Stratified analysis of the Japanese Rhinoconjunctivitis Quality of Life Questionnaire general state by age (≤ 11 years, 12–17 years, and ≥ 18 years) (A), comorbidities (B), and Japanese cedar pollen‐specific immunoglobulin E (< 17.5 UA/mL, ≥ 17.5 UA/mL) (C). [file CLT2-16-e70157-s003.pdf]

Figure S3

(A)

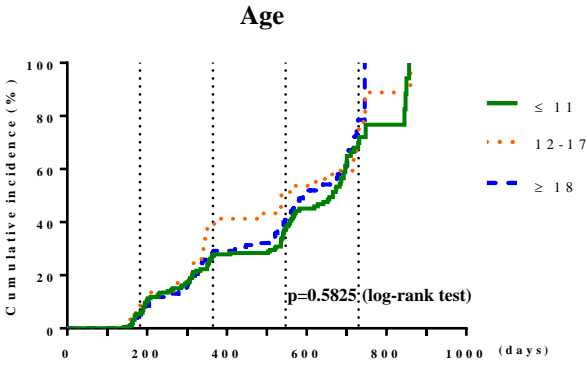

(B)

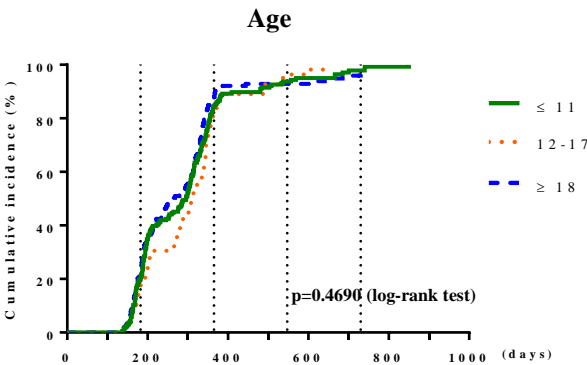

Mono-/Poly-sensitization

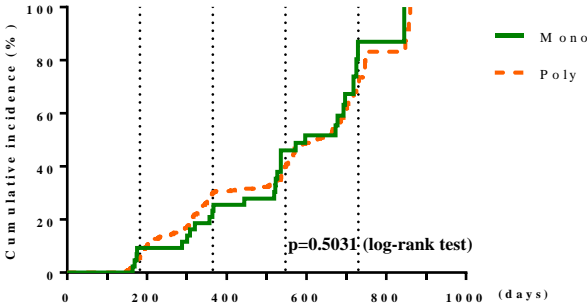

Mono-/Poly-sensitization

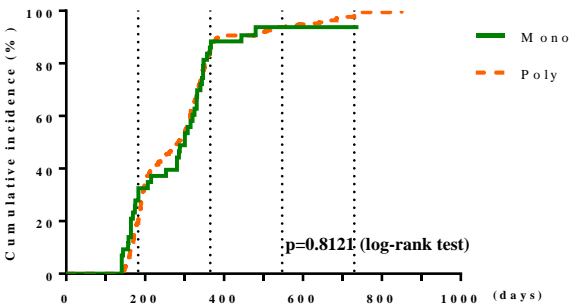

Single/Dual SLIT

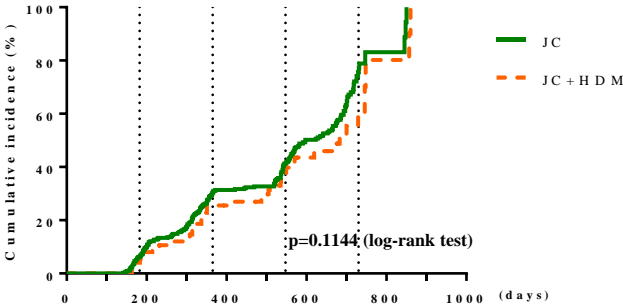

Single/Dual SLIT

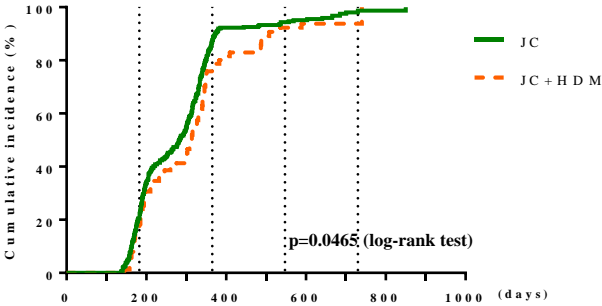

Supplement: Supplementary file 4 — Figure S3: Stratified analysis of cumulative incidence of patients who were recorded as “almost asymptomatic” (A) and “at least one level of improvement” (B) for Japanese cedar pollinosis symptom severity after receiving Japanese cedar pollen sublingual immunotherapy tablets. [file CLT2-16-e70157-s007.pdf]
