# Supplementary material for: Real‐Life Safety of Japanese Cedar Pollen Sublingual Immunotherapy Tablets: A Post‐Marketing Survey
Source: Clin Transl Allergy. 2026 Feb 13;16(2):e70157. doi: 10.1002/clt2.70157 (PMC12904777; doi:10.1002/clt2.70157)
Supplement: Supplementary file 5 — Table S1: Frequency and type of adverse drug reactions: Safety analysis set (n = 516). [file CLT2-16-e70157-s004.docx]

**Table S1. Frequency and type of adverse drug reactions: Safety analysis set (n=516)**

| Post-marketing drug use survey | | |
| --- | --- | --- |
| Safety analysis set | 516 | |
| Patients with ADRs | 68 | |
| Proportion of patients with ADRs | 13.18% | |
| Number of observed ADRs | 112 | |
| Item^†^ | Patients, n^‡^ | % |
| Infections and infestations | 1 | 0.19% |
| Sinusitis | 1 | 0.19% |
| Eye disorders | 5 | 0.97% |
| Conjunctivitis allergic | 1 | 0.19% |
| Vision blurred | 1 | 0.19% |
| Eye pruritus | 3 | 0.58% |
| Ear and labyrinth disorders | 12 | 2.33% |
| Ear pruritus | 12 | 2.33% |
| Respiratory, thoracic, and mediastinal disorders | 27 | 5.23% |
| Asthma | 2 | 0.39% |
| Cough | 4 | 0.78% |
| Dysphonia | 1 | 0.19% |
| Rhinitis allergic | 1 | 0.19% |
| Rhinorrhoea | 1 | 0.19% |
| Sneezing | 1 | 0.19% |
| Throat irritation | 15 | 2.91% |
| Throat tightness | 1 | 0.19% |
| Wheezing | 1 | 0.19% |
| Laryngeal discomfort | 1 | 0.19% |
| Oropharyngeal discomfort | 1 | 0.19% |
| Oropharyngeal pain | 2 | 0.39% |
| Nasal pruritus | 1 | 0.19% |
| Gastrointestinal disorders | 28 | 5.43% |
| Cheilitis | 1 | 0.19% |
| Diarrhoea | 1 | 0.19% |
| Oral discomfort | 2 | 0.39% |
| Oral mucosal blistering | 2 | 0.39% |
| Oral pain | 4 | 0.78% |
| Stomatitis | 6 | 1.16% |
| Swollen tongue | 1 | 0.19% |
| Oral pruritus | 6 | 1.16% |
| Oesophageal discomfort | 1 | 0.19% |
| Oral mucosal erythema | 3 | 0.58% |
| Lip pruritus | 1 | 0.19% |
| Mouth swelling | 10 | 1.94% |
| Skin and subcutaneous tissue disorders | 14 | 2.71% |
| Dermatitis atopic | 2 | 0.39% |
| Eczema | 2 | 0.39% |
| Erythema | 1 | 0.19% |
| Hyperhidrosis | 1 | 0.19% |
| Papule | 1 | 0.19% |
| Pruritus | 3 | 0.58% |
| Rash | 3 | 0.58% |
| Urticaria | 2 | 0.39% |
| General disorders and administration site conditions | 1 | 0.19% |
| Feeling hot | 1 | 0.19% |
| Malaise | 1 | 0.19% |
| † Based on MedDRA/J version 26.0, the data are listed with SOC and PT. | | |
| ‡ If a patient had an adverse reaction of different PTs in the same SOC, the number of cases in that SOC was counted as 1. ADR, adverse drug reaction; PT, preferred terms; SOC, system organ class | | |
