# Supplementary material for: Real‐Life Safety of Japanese Cedar Pollen Sublingual Immunotherapy Tablets: A Post‐Marketing Survey
Source: Clin Transl Allergy. 2026 Feb 13;16(2):e70157. doi: 10.1002/clt2.70157 (PMC12904777; doi:10.1002/clt2.70157)
Supplement: Supplementary file 6 — Table S2: Frequency and type of adverse drug reactions by patient background (n = 516). [file CLT2-16-e70157-s002.docx]

**Table S2. Frequency and type of adverse drug reactions by patient background (n=516)**

| Variables | | | Safety analysis set |  | Patients with ADRs | | p-value^†^ | |
| --- | --- | --- | --- | --- | --- | --- | --- | --- |
|  |  |  |  |  | (Proportion of patients with ADRs) | | Fisher | χ^2^ |
| Analysis set |  |  | 516 |  | 68 | (13.18) |  |  |
| Sex |  | Male | 279 |  | 30 | (10.75) | p=0.090 |  |
|  |  | Female | 237 |  | 38 | (16.03) |  |  |
| Pregnancy^‡^ |  | No | 234 |  | 38 | (16.24) | p=1.000 |  |
|  |  | Yes | 3 |  | 0 | (0.00) |  |  |
| Breast feeding^‡^ |  | No | 237 |  | 38 | (16.03) |  |  |
|  |  | Yes | 0 |  | 0 | (-) |  |  |
| Age 1 |  | <12 years | 218 |  | 29 | (13.30) |  | p=0.721 |
|  |  | ≥12 to <18 years | 85 |  | 9 | (10.59) |  |  |
|  |  | ≥18 years | 213 |  | 30 | (14.08) |  |  |
| Age 2 | Children | ＜15 years | 284 |  | 38 | (13.38) |  | p=0.062 |
|  | Adults | ≥15 to <65 years | 221 |  | 26 | (11.76) |  |  |
|  | Elderly | ≥65 years | 11 |  | 4 | (36.36) |  |  |
| Age 3 |  | ＜12 years | 218 |  | 29 | (13.30) | p=1.000 |  |
|  |  | ≥12 years | 298 |  | 39 | (13.09) |  |  |
| Age 4 |  | ＜65 years | 505 |  | 64 | (12.67) | p=0.044 |  |
|  |  | ≥65 years | 11 |  | 4 | (36.36) |  |  |
| Age 5 |  | ＜15 years | 284 |  | 38 | (13.38) | p=0.897 |  |
|  |  | ≥15 years | 232 |  | 30 | (12.93) |  |  |
| Admission or Outpatient |  | Admission | 0 |  | 0 | (-) | - |  |
|  |  | Outpatient | 516 |  | 68 | (13.18) |  |  |
| Family history of JC-pollinosis | | No | 83 |  | 6 | (7.23) | p=0.163 |  |
|  |  | Yes | 218 |  | 30 | (13.76) |  |  |
|  |  | Unknown | 215 |  | 32 | (14.88) |  |  |
| Smoking |  | No | 462 |  | 64 | (13.85) | p=0.617 |  |
|  |  | Yes | 9 |  | 0 | (0.00) |  |  |
|  |  | Unknown | 45 |  | 4 | (8.89) |  |  |
| Drinking |  | No | 402 |  | 48 | (11.94) | p=0.040 |  |
|  |  | Yes | 59 |  | 13 | (22.03) |  |  |
|  |  | Unknown | 55 |  | 7 | (12.73) |  |  |
| Medical history | No |  | 465 |  | 56 | (12.04) | p=0.018 |  |
|  | Yes |  | 43 |  | 11 | (25.58) |  |  |
|  | Disease^§^ | Asthma | 18 |  | 6 | (33.33) |  |  |
|  |  | Atopic dermatitis | 2 |  | 0 | (0.00) |  |  |
|  |  | Urticaria | 5 |  | 2 | (40.00) |  |  |
|  |  | Allergic rhinitis | 2 |  | 1 | (50.00) |  |  |
|  |  | Food allergy | 9 |  | 2 | (22.22) |  |  |
|  |  | Hypertension | 0 |  | 0 | (-) |  |  |
|  |  | Dyslipidemia | 0 |  | 0 | (-) |  |  |
|  |  | Diabetes mellitus | 0 |  | 0 | (-) |  |  |
|  |  | Sinusitis | 3 |  | 1 | (33.33) |  |  |
|  |  | Others | 12 |  | 1 | (8.33) |  |  |
|  | Unknown |  | 8 |  | 1 | (12.50) |  |  |
| Comorbidities | No |  | 202 |  | 17 | (8.42) | p=0.011 |  |
|  | Yes |  | 306 |  | 50 | (16.34) |  |  |
|  | Disease^§^ | Asthma | 58 |  | 14 | (24.14) |  |  |
|  |  | Atopic dermatitis | 43 |  | 7 | (16.28) |  |  |
|  |  | Urticaria | 4 |  | 0 | (0.00) |  |  |
|  |  | Allergic rhinitis | 253 |  | 40 | (15.81) |  |  |
|  |  | Food allergy | 21 |  | 5 | (23.81) |  |  |
|  |  | Hypertension | 5 |  | 3 | (60.00) |  |  |
|  |  | Dyslipidemia | 9 |  | 2 | (22.22) |  |  |
|  |  | Diabetes mellitus | 3 |  | 0 | (0.00) |  |  |
|  |  | Sinusitis | 7 |  | 1 | (14.29) |  |  |
|  |  | Others | 52 |  | 12 | (23.08) |  |  |
|  |  | Allergic conjunctivitis | 20 |  | 3 | (15.00) |  |  |
|  |  | Liver disease | 0 |  | 0 | (-) |  |  |
|  |  | Kidney disease | 1 |  | 1 | (100.00) |  |  |
|  | Unknown |  | 8 |  | 1 | (12.50) |  |  |
| Comorbidities (Liver disease) |  | No | 508 |  | 67 | (13.19) | - |  |
|  |  | Yes | 0 |  | 0 | (-) |  |  |
|  |  | Unknown | 8 |  | 1 | (12.50) |  |  |
| Comorbidities (Kidney disease) |  | No | 507 |  | 66 | (13.02) | p=0.132 |  |
|  |  | Yes | 1 |  | 1 | (100.00) |  |  |
|  |  | Unknown | 8 |  | 1 | (12.50) |  |  |
| Duration of JC-pollinosis |  | <1 year | 25 |  | 3 | (12.00) |  | p=0.821 |
|  |  | ≥1 to <3 years | 55 |  | 7 | (12.73) |  |  |
|  |  | ≥3 to <6 years | 67 |  | 6 | (8.96) |  |  |
|  |  | ≥6 to <11 years | 65 |  | 10 | (15.38) |  |  |
|  |  | ≥11 to <16 years | 14 |  | 2 | (14.29) |  |  |
|  |  | ≥16 to <21 years | 12 |  | 3 | (25.00) |  |  |
|  |  | ≥21 years | 27 |  | 4 | (14.81) |  |  |
|  |  | Unknown | 251 |  | 33 | (13.15) |  |  |
| Severity of JC-pollinosis symptoms (baseline) | | Most severe | 128 |  | 24 | (18.75) |  | p=0.064 |
|  |  | Severe | 241 |  | 23 | (9.54) |  |  |
|  |  | Moderate | 60 |  | 6 | (10.00) |  |  |
|  |  | Mild | 12 |  | 0 | (0.00) |  |  |
|  |  | Almost asymptomatic | 1 |  | 0 | (0.00) |  |  |
|  |  | Unknown | 74 |  | 15 | (20.27) |  |  |
| JC pollen-specific IgE (baseline) |  | <0.35 UA/mL | 0 |  | 0 | (-) |  | p=0.068 |
|  |  | ≥0.35 to <0.7 UA/mL | 6 |  | 1 | (16.67) |  |  |
|  |  | ≥0.7 to <3.5 UA/mL | 38 |  | 2 | (5.26) |  |  |
|  |  | ≥3.5 to <17.5 UA/mL | 124 |  | 15 | (12.10) |  |  |
|  |  | ≥17.5 to <50 UA/mL | 118 |  | 11 | (9.32) |  |  |
|  |  | ≥50 to <100 UA/mL | 91 |  | 9 | (9.89) |  |  |
|  |  | ≥100 UA/mL | 100 |  | 21 | (21.00) |  |  |
|  |  | Unknown | 39 |  | 9 | (23.08) |  |  |
| Symptom score (baseline) | Sneezing | 4+ | 81 |  | 13 | (16.05) |  |  |
|  |  | 3+ | 173 |  | 15 | (8.67) |  |  |
|  |  | 2+ | 105 |  | 13 | (12.38) |  |  |
|  |  | 1+ | 45 |  | 6 | (13.33) |  |  |
|  |  | － | 18 |  | 4 | (22.22) |  |  |
|  | Runny nose | 4+ | 136 |  | 19 | (13.97) |  |  |
|  |  | 3+ | 167 |  | 17 | (10.18) |  |  |
|  |  | 2+ | 90 |  | 11 | (12.22) |  |  |
|  |  | 1+ | 22 |  | 3 | (13.64) |  |  |
|  |  | － | 7 |  | 1 | (14.29) |  |  |
|  | Congested nose | 4+ | 112 |  | 15 | (13.39) |  |  |
|  |  | 3+ | 171 |  | 18 | (10.53) |  |  |
|  |  | 2+ | 93 |  | 11 | (11.83) |  |  |
|  |  | 1+ | 29 |  | 5 | (17.24) |  |  |
|  |  | － | 17 |  | 2 | (11.76) |  |  |
|  | Difficulty in daily life | 4+ | 76 |  | 16 | (21.05) |  |  |
|  |  | 3+ | 167 |  | 17 | (10.18) |  |  |
|  |  | 2+ | 131 |  | 10 | (7.63) |  |  |
|  |  | 1+ | 35 |  | 6 | (17.14) |  |  |
|  |  | － | 13 |  | 2 | (15.38) |  |  |
|  | Nasal pruritus | 3+ | 67 |  | 10 | (14.93) |  |  |
|  |  | 2+ | 148 |  | 15 | (10.14) |  |  |
|  |  | 1+ | 134 |  | 16 | (11.94) |  |  |
|  |  | － | 73 |  | 10 | (13.70) |  |  |
|  | Eye pruritus | 3+ | 117 |  | 15 | (12.82) |  |  |
|  |  | 2+ | 158 |  | 17 | (10.76) |  |  |
|  |  | 1+ | 92 |  | 12 | (13.04) |  |  |
|  |  | － | 55 |  | 7 | (12.73) |  |  |
|  | Epiphora | 3+ | 41 |  | 7 | (17.07) |  |  |
|  |  | 2+ | 113 |  | 14 | (12.39) |  |  |
|  |  | 1+ | 116 |  | 11 | (9.48) |  |  |
|  |  | － | 152 |  | 19 | (12.50) |  |  |
| QoL score (baseline) |  | 4 | 122 |  | 20 | (16.39) |  |  |
|  |  | 3 | 226 |  | 24 | (10.62) |  |  |
|  |  | 2 | 48 |  | 5 | (10.42) |  |  |
|  |  | 1 | 7 |  | 0 | (0.00) |  |  |
|  |  | 0 | 0 |  | 0 | (-) |  |  |
| Allergies other than JC pollen allergens (baseline) | No |  | 54 |  | 5 | (9.26) | p=0.523 |  |
|  | Yes |  | 452 |  | 62 | (13.72) |  |  |
|  | Allergens^§^ | Mite | 289 |  | 37 | (12.80) |  |  |
|  |  | House dust | 237 |  | 28 | (11.81) |  |  |
|  |  | Dog | 69 |  | 7 | (10.14) |  |  |
|  |  | Cat | 94 |  | 14 | (14.89) |  |  |
|  |  | Moth | 36 |  | 3 | (8.33) |  |  |
|  |  | Cypress pollen | 330 |  | 42 | (12.73) |  |  |
|  |  | Orchard grass pollen | 139 |  | 21 | (15.11) |  |  |
|  |  | Ragweed pollen | 102 |  | 18 | (17.65) |  |  |
|  |  | Mugwort pollen | 52 |  | 11 | (21.15) |  |  |
|  |  | Alder pollen | 78 |  | 12 | (15.38) |  |  |
|  |  | Timothy grass pollen | 41 |  | 6 | (14.63) |  |  |
|  |  | Sweet vernal grass pollen | 9 |  | 3 | (33.33) |  |  |
|  |  | White birch pollen | 48 |  | 9 | (18.75) |  |  |
|  |  | Egg white | 7 |  | 2 | (28.57) |  |  |
|  |  | Milk | 8 |  | 2 | (25.00) |  |  |
|  |  | Wheat | 16 |  | 4 | (25.00) |  |  |
|  |  | Peanut | 19 |  | 2 | (10.53) |  |  |
|  |  | Buckwheat | 13 |  | 2 | (15.38) |  |  |
|  |  | Shrimp | 7 |  | 0 | (0.00) |  |  |
|  |  | Crab | 8 |  | 1 | (12.50) |  |  |
|  |  | Apple | 16 |  | 5 | (31.25) |  |  |
|  |  | Kiwi | 15 |  | 3 | (20.00) |  |  |
|  |  | Peach | 4 |  | 1 | (25.00) |  |  |
|  |  | Celery | 0 |  | 0 | (-) |  |  |
|  |  | Tomato | 5 |  | 1 | (20.00) |  |  |
|  |  | Others | 62 |  | 9 | (14.52) |  |  |
|  | Unknown |  | 10 |  | 1 | (10.00) |  |  |
| Average daily dose |  | ≤2,000 JAU | 8 |  | 4 | (50.00) |  | p<0.001 |
|  |  | >2,000 JAU and ≤4,000 JAU | 6 |  | 5 | (83.33) |  |  |
|  |  | >4,000 JAU | 502 |  | 59 | (11.75) |  |  |
|  |  | Unknown | 0 |  | 0 | (-) |  |  |
| Total dose |  | ≤15,000 JAU | 5 |  | 2 | (40.00) |  |  |
|  |  | >15,000 to ≤50,000 JAU | 1 |  | 1 | (100.00) |  |  |
|  |  | >50,000 to ≤120,000 JAU | 3 |  | 1 | (33.33) |  |  |
|  |  | >120,000 to ≤880,000 JAU | 34 |  | 10 | (29.41) |  |  |
|  |  | >880,000 to ≤1,330,000 JAU | 17 |  | 3 | (17.65) |  |  |
|  |  | >1,330,000 to ≤1,780,000 JAU | 22 |  | 3 | (13.64) |  |  |
|  |  | >1,780,000 to ≤2,680,000 JAU | 157 |  | 19 | (12.10) |  |  |
|  |  | >2,680,000 to ≤3,130,000 JAU | 90 |  | 9 | (10.00) |  |  |
|  |  | >3,130,000 to ≤3,580,000 JAU | 166 |  | 15 | (9.04) |  |  |
|  |  | >3,580,000 JAU | 21 |  | 5 | (23.81) |  |  |
|  |  | Unknown | 0 |  | 0 | (-) |  |  |
| Treatment duration |  | ≤7 days | 5 |  | 2 | (40.00) |  | p=0.006 |
|  |  | >7 to ≤14 days | 1 |  | 1 | (100.00) |  |  |
|  |  | >14 to ≤28 days | 2 |  | 0 | (0.00) |  |  |
|  |  | >28 to ≤180 days | 33 |  | 10 | (30.30) |  |  |
|  |  | >180 to ≤270 days | 16 |  | 2 | (12.50) |  |  |
|  |  | >270 to ≤360 days | 20 |  | 3 | (15.00) |  |  |
|  |  | >360 to ≤540 days | 154 |  | 19 | (12.34) |  |  |
|  |  | >540 to ≤630 days | 90 |  | 8 | (8.89) |  |  |
|  |  | >630 to ≤720 days | 173 |  | 18 | (10.40) |  |  |
|  |  | >720 days | 22 |  | 5 | (22.73) |  |  |
|  |  | Unknown | 0 |  | 0 | (-) |  |  |
| Previous treatment for JC-pollinosis | | No | 132 |  | 9 | (6.82) | p=0.026 |  |
|  |  | Yes | 305 |  | 45 | (14.75) |  |  |
|  |  | Unknown | 79 |  | 14 | (17.72) |  |  |
| Concomitant drugs^¶^ | | No | 150 |  | 15 | (10.00) | p=0.198 |  |
|  |  | Yes | 366 |  | 53 | (14.48) |  |  |
| Allergen immunotherapy (baseline) | | No | 464 |  | 66 | (14.22) | p=0.031 |  |
|  |  | Yes | 52 |  | 2 | (3.85) |  |  |
| Allergen immunotherapy^‡^ (after administration) | | No | 416 |  | 58 | (13.94) | p=0.328 |  |
|  |  | Yes | 100 |  | 10 | (10.00) |  |  |
| Treatment for JC-pollinosis (baseline) | | No | 503 |  | 67 | (13.32) | p=1.000 |  |
|  |  | Yes | 2 |  | 0 | (0.00) |  |  |
|  |  | Unknown | 11 |  | 1 | (9.09) |  |  |
| Treatment for JC-pollinosis^‡^ (after administration) | | No | 502 |  | 66 | (13.15) | p=0.348 |  |
|  |  | Yes | 3 |  | 1 | (33.33) |  |  |
|  |  | Unknown | 11 |  | 1 | (9.09) |  |  |
| Treatment for conditions other than JC-pollinosis^‡,\|\|^ (after administration) | | No | 503 |  | 66 | (13.12) | p=0.248 |  |
|  |  | Yes | 2 |  | 1 | (50.00) |  |  |
|  |  | Unknown | 11 |  | 1 | (9.09) |  |  |

† Fisher's exact probability tests were performed for two-category analysis items, and χ^2^ tests were performed for three or more categories of analysis items

‡ Aggregate data from both Season 1 and Season 2

§Multiple answers possible

¶ Concomitant drugs for which the reason for use was “adverse event treatment” were excluded

|| Surgical treatments for which the reason for use was “adverse event treatment” were excluded

ADR, adverse drug reaction; IgE, immunoglobulin E; JAU, Japanese allergy unit; JC, Japanese cedar; QoL, quality of life
