# Supplementary material for: Real‐Life Safety of Japanese Cedar Pollen Sublingual Immunotherapy Tablets: A Post‐Marketing Survey
Source: Clin Transl Allergy. 2026 Feb 13;16(2):e70157. doi: 10.1002/clt2.70157 (PMC12904777; doi:10.1002/clt2.70157)
Supplement: Supplementary file 7 — Table S3: Improvement rate (symptom severity of cedar pollen allergy) by patient background in effectiveness analysis set (n = 469). [file CLT2-16-e70157-s005.docx]

**Table S3. Improvement rate (symptom severity of cedar pollen allergy) by patient background in effectiveness analysis set (n=469)**

| Variables | | | Season 1 | | | | | Season 2 | | | | |
| --- | --- | --- | --- | --- | --- | --- | --- | --- | --- | --- | --- | --- |
|  |  |  | Effectiveness analysis set | Number of cases improved | Number of cases not improved | p-value^†^ | | Effectiveness analysis set | Number of cases improved | Number of cases not improved | p-value^†^ | |
|  |  |  |  | (Improvement rate) | (Non-improvement rate) | Fisher | χ^2^ |  | (Improvement rate) | (Non-improvement rate) | Fisher | χ^2^ |
| Analysis set |  |  | 395 | 372(94.18) | 23(5.82) |  |  | 346 | 335(96.82) | 11(3.18) |  |  |
| Sex |  | Male | 213 | 200(93.90) | 13(6.10) | p=0.833 |  | 189 | 182(96.30) | 7(3.70) | p=0.760 |  |
|  |  | Female | 182 | 172(94.51) | 10(5.49) |  |  | 157 | 153(97.45) | 4(2.55) |  |  |
| Pregnancy^‡^ |  | No | 182 | 172(94.51) | 10(5.49) | - |  | 157 | 153(97.45) | 4(2.55) | - |  |
|  |  | Yes | 0 | 0(-) | 0(-) |  |  | 0 | 0(-) | 0(-) |  |  |
| Breastfeeding^‡^ |  | No | 182 | 172(94.51) | 10(5.49) |  |  | 157 | 153(97.45) | 4(2.55) |  |  |
|  |  | Yes | 0 | 0(-) | 0(-) |  |  | 0 | 0(-) | 0(-) |  |  |
| Age 1 |  | ≤11 years | 179 | 171(95.53) | 8(4.47) |  | p=0.118 | 170 | 165(97.06) | 5(2.94) |  | p=0.803 |
|  |  | 12–17 years | 61 | 54(88.52) | 7(11.48) |  |  | 48 | 47(97.92) | 1(2.08) |  |  |
|  |  | ≥18 years | 155 | 147(94.84) | 8(5.16) |  |  | 128 | 123(96.09) | 5(3.91) |  |  |
| Age 2 | Children | <15 years | 227 | 212(93.39) | 15(6.61) |  | p=0.405 | 207 | 201(97.10) | 6(2.90) |  | p=0.044 |
|  | Adults | ≥15 to <65 years | 161 | 154(95.65) | 7(4.35) |  |  | 135 | 131(97.04) | 4(2.96) |  |  |
|  | Elderly | ≥65 years | 7 | 6(85.71) | 1(14.29) |  |  | 4 | 3(75.00) | 1(25.00) |  |  |
| Age 3 |  | <12 years | 179 | 171(95.53) | 8(4.47) | p=0.389 |  | 170 | 165(97.06) | 5(2.94) | p=1.000 |  |
|  |  | ≥12 years | 216 | 201(93.06) | 15(6.94) |  |  | 176 | 170(96.59) | 6(3.41) |  |  |
| Age 4 |  | <65 years | 388 | 366(94.33) | 22(5.67) | p=0.345 |  | 342 | 332(97.08) | 10(2.92) | p=0.122 |  |
|  |  | ≥65 years | 7 | 6(85.71) | 1(14.29) |  |  | 4 | 3(75.00) | 1(25.00) |  |  |
| Age 5 |  | <15 years | 227 | 212(93.39) | 15(6.61) | p=0.518 |  | 207 | 201(97.10) | 6(2.90) | p=0.761 |  |
|  |  | ≥15 years | 168 | 160(95.24) | 8(4.76) |  |  | 139 | 134(96.40) | 5(3.60) |  |  |
| Admission or Outpatient |  | Admission | 0 | 0(-) | 0(-) | - |  | 0 | 0(-) | 0(-) | - |  |
|  |  | Outpatient | 395 | 372(94.18) | 23(5.82) |  |  | 346 | 335(96.82) | 11(3.18) |  |  |
| Family history of JC-pollinosis | | No | 69 | 66(95.65) | 3(4.35) | p=1.000 |  | 62 | 59(95.16) | 3(4.84) | p=0.078 |  |
|  |  | Yes | 171 | 163(95.32) | 8(4.68) |  |  | 148 | 147(99.32) | 1(0.68) |  |  |
|  |  | Unknown | 155 | 143(92.26) | 12(7.74) |  |  | 136 | 129(94.85) | 7(5.15) |  |  |
| Smoking |  | No | 357 | 339(94.96) | 18(5.04) | p=0.051 |  | 319 | 310(97.18) | 9(2.82) | p=1.000 |  |
|  |  | Yes | 7 | 5(71.43) | 2(28.57) |  |  | 4 | 4(100.00) | 0(0.00) |  |  |
|  |  | Unknown | 31 | 28(90.32) | 3(9.68) |  |  | 23 | 21(91.30) | 2(8.70) |  |  |
| Drinking |  | No | 314 | 297(94.59) | 17(5.41) | p=0.720 |  | 285 | 277(97.19) | 8(2.81) | p=1.000 |  |
|  |  | Yes | 43 | 40(93.02) | 3(6.98) |  |  | 34 | 34(100.00) | 0(0.00) |  |  |
|  |  | Unknown | 38 | 35(92.11) | 3(7.89) |  |  | 27 | 24(88.89) | 3(11.11) |  |  |
| Medical history | No |  | 356 | 337(94.66) | 19(5.34) | p=0.129 |  | 315 | 305(96.83) | 10(3.17) | p=0.601 |  |
|  | Yes |  | 34 | 30(88.24) | 4(11.76) |  |  | 27 | 26(96.30) | 1(3.70) |  |  |
|  | Disease^§^ | Asthma | 13 | 10(76.92) | 3(23.08) |  |  | 11 | 10(90.91) | 1(9.09) |  |  |
|  |  | Atopic dermatitis | 0 | 0(-) | 0(-) |  |  | 0 | 0(-) | 0(-) |  |  |
|  |  | Urticaria | 4 | 3(75.00) | 1(25.00) |  |  | 4 | 4(100.00) | 0(0.00) |  |  |
|  |  | Allergic rhinitis | 0 | 0(-) | 0(-) |  |  | 0 | 0(-) | 0(-) |  |  |
|  |  | Food allergy | 8 | 7(87.50) | 1(12.50) |  |  | 7 | 7(100.00) | 0(0.00) |  |  |
|  |  | Hypertension | 0 | 0(-) | 0(-) |  |  | 0 | 0(-) | 0(-) |  |  |
|  |  | Dyslipidemia | 0 | 0(-) | 0(-) |  |  | 0 | 0(-) | 0(-) |  |  |
|  |  | Diabetes mellitus | 0 | 0(-) | 0(-) |  |  | 0 | 0(-) | 0(-) |  |  |
|  |  | Sinusitis | 3 | 3(100.00) | 0(0.00) |  |  | 2 | 2(100.00) | 0(0.00) |  |  |
|  |  | Others | 12 | 10(83.33) | 2(16.67) |  |  | 7 | 6(85.71) | 1(14.29) |  |  |
|  | Unknown |  | 5 | 5(100.00) | 0(0.00) |  |  | 4 | 4(100.00) | 0(0.00) |  |  |
| Comorbidities | No |  | 167 | 162(97.01) | 5(2.99) | p=0.049 |  | 139 | 136(97.84) | 3(2.16) | p=0.535 |  |
|  | Yes |  | 223 | 205(91.93) | 18(8.07) |  |  | 203 | 195(96.06) | 8(3.94) |  |  |
|  | Disease^§^ | Asthma | 48 | 47(97.92) | 1(2.08) |  |  | 43 | 42(97.67) | 1(2.33) |  |  |
|  |  | Atopic dermatitis | 33 | 30(90.91) | 3(9.09) |  |  | 25 | 25(100.00) | 0(0.00) |  |  |
|  |  | Urticaria | 4 | 4(100.00) | 0(0.00) |  |  | 4 | 4(100.00) | 0(0.00) |  |  |
|  |  | Allergic rhinitis | 187 | 173(92.51) | 14(7.49) |  |  | 174 | 166(95.40) | 8(4.60) |  |  |
|  |  | Food allergy | 15 | 15(100.00) | 0(0.00) |  |  | 12 | 12(100.00) | 0(0.00) |  |  |
|  |  | Hypertension | 2 | 0(0.00) | 2(100.00) |  |  | 3 | 3(100.00) | 0(0.00) |  |  |
|  |  | Dyslipidemia | 6 | 5(83.33) | 1(16.67) |  |  | 7 | 7(100.00) | 0(0.00) |  |  |
|  |  | Diabetes mellitus | 3 | 3(100.00) | 0(0.00) |  |  | 2 | 2(100.00) | 0(0.00) |  |  |
|  |  | Sinusitis | 5 | 4(80.00) | 1(20.00) |  |  | 5 | 4(80.00) | 1(20.00) |  |  |
|  |  | Others | 39 | 35(89.74) | 4(10.26) |  |  | 33 | 32(96.97) | 1(3.03) |  |  |
|  |  | Allergic conjunctivitis | 18 | 17(94.44) | 1(5.56) |  |  | 16 | 15(93.75) | 1(6.25) |  |  |
|  |  | Liver disease | 0 | 0(-) | 0(-) |  |  | 0 | 0(-) | 0(-) |  |  |
|  |  | Kidney disease | 0 | 0(-) | 0(-) |  |  | 0 | 0(-) | 0(-) |  |  |
|  | Unknown |  | 5 | 5(100.00) | 0(0.00) |  |  | 4 | 4(100.00) | 0(0.00) |  |  |
| Comorbidities (Liver disease) |  | No | 390 | 367(94.10) | 23(5.90) | - |  | 342 | 331(96.78) | 11(3.22) | - |  |
|  |  | Yes | 0 | 0(-) | 0(-) |  |  | 0 | 0(-) | 0(-) |  |  |
|  |  | Unknown | 5 | 5(100.00) | 0(0.00) |  |  | 4 | 4(100.00) | 0(0.00) |  |  |
| Comorbidities (Kidney disease) | | No | 390 | 367(94.10) | 23(5.90) | - |  | 342 | 331(96.78) | 11(3.22) | - |  |
|  |  | Yes | 0 | 0(-) | 0(-) |  |  | 0 | 0(-) | 0(-) |  |  |
|  |  | Unknown | 5 | 5(100.00) | 0(0.00) |  |  | 4 | 4(100.00) | 0(0.00) |  |  |
| Duration of JC-pollinosis | | <1 year | 21 | 19(90.48) | 2(9.52) |  | p=0.602 | 18 | 17(94.44) | 1(5.56) |  | p=0.761 |
|  |  | ≥1 to <3 years | 45 | 41(91.11) | 4(8.89) |  |  | 42 | 42(100.00) | 0(0.00) |  |  |
|  |  | ≥3 to <6 years | 57 | 55(96.49) | 2(3.51) |  |  | 55 | 54(98.18) | 1(1.82) |  |  |
|  |  | ≥6 to <11 years | 51 | 48(94.12) | 3(5.88) |  |  | 40 | 39(97.50) | 1(2.50) |  |  |
|  |  | ≥11 to <16 years | 12 | 12(100.00) | 0(0.00) |  |  | 9 | 9(100.00) | 0(0.00) |  |  |
|  |  | ≥16 to <21 years | 9 | 9(100.00) | 0(0.00) |  |  | 8 | 8(100.00) | 0(0.00) |  |  |
|  |  | ≥21 years | 24 | 21(87.50) | 3(12.50) |  |  | 20 | 20(100.00) | 0(0.00) |  |  |
|  |  | Unknown | 176 | 167(94.89) | 9(5.11) |  |  | 154 | 146(94.81) | 8(5.19) |  |  |
| Severity of JC-pollinosis symptoms (baseline) | | Most severe | 108 | 104(96.30) | 4(3.70) |  | p<0.001 | 95 | 95(100.00) | 0(0.00) |  | p<0.001 |
|  |  | Severe | 222 | 215(96.85) | 7(3.15) |  |  | 201 | 196(97.51) | 5(2.49) |  |  |
|  |  | Moderate | 54 | 49(90.74) | 5(9.26) |  |  | 41 | 40(97.56) | 1(2.44) |  |  |
|  |  | Mild | 10 | 4(40.00) | 6(60.00) |  |  | 8 | 4(50.00) | 4(50.00) |  |  |
|  |  | no symptoms | 1 | 0(0.00) | 1(100.00) |  |  | 1 | 0(0.00) | 1(100.00) |  |  |
|  |  | Unknown | 0 | 0(-) | 0(-) |  |  | 0 | 0(-) | 0(-) |  |  |
| JC pollen-specific IgE (baseline) | | <0.35 UA/mL | 0 | 0(-) | 0(-) |  | p=0.525 | 0 | 0(-) | 0(-) |  | p=0.657 |
|  |  | ≥0.35 to <0.7 UA/mL | 5 | 5(100.00) | 0(0.00) |  |  | 5 | 5(100.00) | 0(0.00) |  |  |
|  |  | ≥0.7 to <3.5 UA/mL | 23 | 23(100.00) | 0(0.00) |  |  | 20 | 20(100.00) | 0(0.00) |  |  |
|  |  | ≥3.5 to <17.5 UA/mL | 95 | 87(91.58) | 8(8.42) |  |  | 77 | 74(96.10) | 3(3.90) |  |  |
|  |  | ≥17.5 to <50 UA/mL | 91 | 87(95.60) | 4(4.40) |  |  | 80 | 79(98.75) | 1(1.25) |  |  |
|  |  | ≥50 to <100 UA/mL | 74 | 71(95.95) | 3(4.05) |  |  | 65 | 63(96.92) | 2(3.08) |  |  |
|  |  | ≥100 UA/mL | 79 | 73(92.41) | 6(7.59) |  |  | 72 | 68(94.44) | 4(5.56) |  |  |
|  |  | Unknown | 28 | 26(92.86) | 2(7.14) |  |  | 27 | 26(96.30) | 1(3.70) |  |  |
| Symptom score (baseline) | Sneezing | 4+ | 74 | 72(97.30) | 2(2.70) |  |  | 63 | 63(100.00) | 0(0.00) |  |  |
|  |  | 3+ | 154 | 148(96.10) | 6(3.90) |  |  | 135 | 131(97.04) | 4(2.96) |  |  |
|  |  | 2+ | 97 | 91(93.81) | 6(6.19) |  |  | 85 | 84(98.82) | 1(1.18) |  |  |
|  |  | 1+ | 35 | 30(85.71) | 5(14.29) |  |  | 32 | 29(90.63) | 3(9.38) |  |  |
|  |  | － | 16 | 12(75.00) | 4(25.00) |  |  | 16 | 13(81.25) | 3(18.75) |  |  |
|  | Runny nose | 4+ | 120 | 115(95.83) | 5(4.17) |  |  | 105 | 104(99.05) | 1(0.95) |  |  |
|  |  | 3+ | 150 | 144(96.00) | 6(4.00) |  |  | 135 | 131(97.04) | 4(2.96) |  |  |
|  |  | 2+ | 82 | 76(92.68) | 6(7.32) |  |  | 70 | 68(97.14) | 2(2.86) |  |  |
|  |  | 1+ | 18 | 16(88.89) | 2(11.11) |  |  | 16 | 15(93.75) | 1(6.25) |  |  |
|  |  | － | 6 | 2(33.33) | 4(66.67) |  |  | 5 | 2(40.00) | 3(60.00) |  |  |
|  | Congested nose | 4+ | 96 | 89(92.71) | 7(7.29) |  |  | 88 | 83(94.32) | 5(5.68) |  |  |
|  |  | 3+ | 153 | 148(96.73) | 5(3.27) |  |  | 134 | 133(99.25) | 1(0.75) |  |  |
|  |  | 2+ | 85 | 80(94.12) | 5(5.88) |  |  | 75 | 73(97.33) | 2(2.67) |  |  |
|  |  | 1+ | 26 | 24(92.31) | 2(7.69) |  |  | 22 | 21(95.45) | 1(4.55) |  |  |
|  |  | － | 16 | 12(75.00) | 4(25.00) |  |  | 12 | 10(83.33) | 2(16.67) |  |  |
|  | Difficulty in daily life | 4+ | 64 | 59(92.19) | 5(7.81) |  |  | 56 | 54(96.43) | 2(3.57) |  |  |
|  |  | 3+ | 150 | 150(100.00) | 0(0.00) |  |  | 137 | 136(99.27) | 1(0.73) |  |  |
|  |  | 2+ | 122 | 114(93.44) | 8(6.56) |  |  | 108 | 104(96.30) | 4(3.70) |  |  |
|  |  | 1+ | 32 | 27(84.38) | 5(15.63) |  |  | 24 | 22(91.67) | 2(8.33) |  |  |
|  |  | － | 8 | 3(37.50) | 5(62.50) |  |  | 6 | 4(66.67) | 2(33.33) |  |  |
|  | Nasal pruritus | 3+ | 60 | 55(91.67) | 5(8.33) |  |  | 48 | 47(97.92) | 1(2.08) |  |  |
|  |  | 2+ | 135 | 130(96.30) | 5(3.70) |  |  | 118 | 113(95.76) | 5(4.24) |  |  |
|  |  | 1+ | 118 | 114(96.61) | 4(3.39) |  |  | 108 | 108(100.00) | 0(0.00) |  |  |
|  |  | － | 63 | 54(85.71) | 9(14.29) |  |  | 57 | 52(91.23) | 5(8.77) |  |  |
|  | Eye pruritus | 3+ | 105 | 97(92.38) | 8(7.62) |  |  | 90 | 86(95.56) | 4(4.44) |  |  |
|  |  | 2+ | 142 | 137(96.48) | 5(3.52) |  |  | 127 | 123(96.85) | 4(3.15) |  |  |
|  |  | 1+ | 83 | 80(96.39) | 3(3.61) |  |  | 74 | 72(97.30) | 2(2.70) |  |  |
|  |  | － | 46 | 39(84.78) | 7(15.22) |  |  | 40 | 39(97.50) | 1(2.50) |  |  |
|  | Epiphora | 3+ | 38 | 34(89.47) | 4(10.53) |  |  | 33 | 31(93.94) | 2(6.06) |  |  |
|  |  | 2+ | 98 | 96(97.96) | 2(2.04) |  |  | 86 | 86(100.00) | 0(0.00) |  |  |
|  |  | 1+ | 106 | 101(95.28) | 5(4.72) |  |  | 93 | 89(95.70) | 4(4.30) |  |  |
|  |  | － | 134 | 122(91.04) | 12(8.96) |  |  | 119 | 114(95.80) | 5(4.20) |  |  |
| QoL score (baseline) | | 4 | 107 | 100(93.46) | 7(6.54) |  |  | 89 | 87(97.75) | 2(2.25) |  |  |
|  |  | 3 | 204 | 199(97.55) | 5(2.45) |  |  | 187 | 180(96.26) | 7(3.74) |  |  |
|  |  | 2 | 43 | 36(83.72) | 7(16.28) |  |  | 36 | 35(97.22) | 1(2.78) |  |  |
|  |  | 1 | 5 | 2(40.00) | 3(60.00) |  |  | 4 | 3(75.00) | 1(25.00) |  |  |
|  |  | 0 | 0 | 0(-) | 0(-) |  |  | 0 | 0(-) | 0(-) |  |  |
| Allergies other than JC pollen allergens (baseline) | No |  | 43 | 40(93.02) | 3(6.98) | p=0.731 |  | 38 | 36(94.74) | 2(5.26) | p=0.353 |  |
|  | Yes |  | 344 | 324(94.19) | 20(5.81) |  |  | 302 | 293(97.02) | 9(2.98) |  |  |
|  | Allergens^§^ | Mite | 219 | 204(93.15) | 15(6.85) |  |  | 193 | 187(96.89) | 6(3.11) |  |  |
|  |  | House dust | 182 | 168(92.31) | 14(7.69) |  |  | 156 | 153(98.08) | 3(1.92) |  |  |
|  |  | Dog | 58 | 53(91.38) | 5(8.62) |  |  | 48 | 47(97.92) | 1(2.08) |  |  |
|  |  | Cat | 81 | 75(92.59) | 6(7.41) |  |  | 66 | 65(98.48) | 1(1.52) |  |  |
|  |  | Moth | 28 | 24(85.71) | 4(14.29) |  |  | 25 | 23(92.00) | 2(8.00) |  |  |
|  |  | Cypress pollen | 254 | 240(94.49) | 14(5.51) |  |  | 221 | 217(98.19) | 4(1.81) |  |  |
|  |  | Orchard grass pollen | 111 | 105(94.59) | 6(5.41) |  |  | 98 | 97(98.98) | 1(1.02) |  |  |
|  |  | Ragweed pollen | 80 | 73(91.25) | 7(8.75) |  |  | 68 | 65(95.59) | 3(4.41) |  |  |
|  |  | Mugwort pollen | 41 | 38(92.68) | 3(7.32) |  |  | 35 | 34(97.14) | 1(2.86) |  |  |
|  |  | Alder pollen | 64 | 62(96.88) | 2(3.13) |  |  | 52 | 50(96.15) | 2(3.85) |  |  |
|  |  | Timothy grass pollen | 35 | 34(97.14) | 1(2.86) |  |  | 27 | 27(100.00) | 0(0.00) |  |  |
|  |  | Sweet vernal grass pollen | 6 | 6(100.00) | 0(0.00) |  |  | 5 | 5(100.00) | 0(0.00) |  |  |
|  |  | White birch pollen | 39 | 38(97.44) | 1(2.56) |  |  | 29 | 29(100.00) | 0(0.00) |  |  |
|  |  | Egg white | 5 | 4(80.00) | 1(20.00) |  |  | 5 | 5(100.00) | 0(0.00) |  |  |
|  |  | Milk | 6 | 6(100.00) | 0(0.00) |  |  | 4 | 4(100.00) | 0(0.00) |  |  |
|  |  | Wheat | 11 | 11(100.00) | 0(0.00) |  |  | 9 | 9(100.00) | 0(0.00) |  |  |
|  |  | Peanut | 15 | 15(100.00) | 0(0.00) |  |  | 12 | 12(100.00) | 0(0.00) |  |  |
|  |  | Buckwheat | 11 | 11(100.00) | 0(0.00) |  |  | 8 | 8(100.00) | 0(0.00) |  |  |
|  |  | Shrimp | 4 | 4(100.00) | 0(0.00) |  |  | 4 | 4(100.00) | 0(0.00) |  |  |
|  |  | Crab | 6 | 5(83.33) | 1(16.67) |  |  | 3 | 3(100.00) | 0(0.00) |  |  |
|  |  | Apple | 9 | 9(100.00) | 0(0.00) |  |  | 9 | 9(100.00) | 0(0.00) |  |  |
|  |  | Kiwi | 10 | 10(100.00) | 0(0.00) |  |  | 8 | 8(100.00) | 0(0.00) |  |  |
|  |  | Peach | 2 | 2(100.00) | 0(0.00) |  |  | 2 | 2(100.00) | 0(0.00) |  |  |
|  |  | Celery | 0 | 0(-) | 0(-) |  |  | 0 | 0(-) | 0(-) |  |  |
|  |  | Tomato | 3 | 3(100.00) | 0(0.00) |  |  | 2 | 2(100.00) | 0(0.00) |  |  |
|  |  | Others | 46 | 45(97.83) | 1(2.17) |  |  | 40 | 38(95.00) | 2(5.00) |  |  |
|  | Unknown |  | 8 | 8(100.00) | 0(0.00) |  |  | 6 | 6(100.00) | 0(0.00) |  |  |
| Average Daily Dose | | ≤2,000 JAU | 1 | 1(100.00) | 0(0.00) |  | p=0.855 | 1 | 1(100.00) | 0(0.00) |  | p=0.936 |
|  |  | 2,000 JAU＜and ≤4,000 JAU | 4 | 4(100.00) | 0(0.00) |  |  | 3 | 3(100.00) | 0(0.00) |  |  |
|  |  | >4,000 JAU＜ | 390 | 367(94.10) | 23(5.90) |  |  | 342 | 331(96.78) | 11(3.22) |  |  |
|  |  | Unknown | 0 | 0(-) | 0(-) |  |  | 0 | 0(-) | 0(-) |  |  |
| Total dose |  | ≤15,000 JAU | 0 | 0(-) | 0(-) |  |  | 0 | 0(-) | 0(-) |  |  |
|  |  | >15,000 to ≤50,000 JAU | 0 | 0(-) | 0(-) |  |  | 0 | 0(-) | 0(-) |  |  |
|  |  | >50,000 to ≤120,000 JAU | 0 | 0(-) | 0(-) |  |  | 0 | 0(-) | 0(-) |  |  |
|  |  | >120,000 to ≤880,000 JAU | 5 | 5(100.00) | 0(0.00) |  |  | 0 | 0(-) | 0(-) |  |  |
|  |  | >880,000 to ≤1,330,000 JAU | 8 | 8(100.00) | 0(0.00) |  |  | 1 | 1(100.00) | 0(0.00) |  |  |
|  |  | >1,330,000 to ≤1,780,000 JAU | 18 | 15(83.33) | 3(16.67) |  |  | 3 | 3(100.00) | 0(0.00) |  |  |
|  |  | >1,780,000 to ≤2,680,000 JAU | 120 | 110(91.67) | 10(8.33) |  |  | 104 | 103(99.04) | 1(0.96) |  |  |
|  |  | >2,680,000 to ≤3,130,000 JAU | 80 | 76(95.00) | 4(5.00) |  |  | 76 | 72(94.74) | 4(5.26) |  |  |
|  |  | >3,130,000 to ≤3,580,000 JAU | 145 | 139(95.86) | 6(4.14) |  |  | 141 | 137(97.16) | 4(2.84) |  |  |
|  |  | >3,580,000 JAU | 19 | 19(100.00) | 0(0.00) |  |  | 21 | 19(90.48) | 2(9.52) |  |  |
|  |  | Unknown | 0 | 0(-) | 0(-) |  |  | 0 | 0(-) | 0(-) |  |  |
| Treatment duration | | ≤7 days | 0 | 0(-) | 0(-) |  | p=0.202 | 0 | 0(-) | 0(-) |  | p=0.150 |
|  |  | >7 to ≤14 days | 0 | 0(-) | 0(-) |  |  | 0 | 0(-) | 0(-) |  |  |
|  |  | >14 to ≤28 days | 0 | 0(-) | 0(-) |  |  | 0 | 0(-) | 0(-) |  |  |
|  |  | >28 to ≤180 days | 4 | 4(100.00) | 0(0.00) |  |  | 0 | 0(-) | 0(-) |  |  |
|  |  | >180 to ≤270 days | 7 | 7(100.00) | 0(0.00) |  |  | 0 | 0(-) | 0(-) |  |  |
|  |  | >270 to ≤360 days | 17 | 14(82.35) | 3(17.65) |  |  | 0 | 0(-) | 0(-) |  |  |
|  |  | >360 to ≤540 days | 120 | 110(91.67) | 10(8.33) |  |  | 101 | 100(99.01) | 1(0.99) |  |  |
|  |  | >540 to ≤630 days | 79 | 75(94.94) | 4(5.06) |  |  | 75 | 71(94.67) | 4(5.33) |  |  |
|  |  | >630 to ≤720 days | 149 | 143(95.97) | 6(4.03) |  |  | 148 | 144(97.30) | 4(2.70) |  |  |
|  |  | >720 days | 19 | 19(100.00) | 0(0.00) |  |  | 22 | 20(90.91) | 2(9.09) |  |  |
|  |  | Unknown | 0 | 0(-) | 0(-) |  |  | 0 | 0(-) | 0(-) |  |  |
| Previous treatment for JC-pollinosis | | No | 101 | 97(96.04) | 4(3.96) | p=0.454 |  | 90 | 86(95.56) | 4(4.44) | p=0.458 |  |
|  |  | Yes | 250 | 234(93.60) | 16(6.40) |  |  | 215 | 210(97.67) | 5(2.33) |  |  |
|  |  | Unknown | 44 | 41(93.18) | 3(6.82) |  |  | 41 | 39(95.12) | 2(4.88) |  |  |
| Concomitant drugs^¶^ | | No | 270 | 256(94.81) | 14(5.19) | p=0.489 |  | 130 | 128(98.46) | 2(1.54) | p=0.220 |  |
|  |  | Yes | 125 | 116(92.80) | 9(7.20) |  |  | 216 | 207(95.83) | 9(4.17) |  |  |
| Allergen immunotherapy (baseline) | | No | 351 | 336(95.73) | 15(4.27) | p=0.002 |  | 309 | 300(97.09) | 9(2.91) | p=0.333 |  |
|  |  | Yes | 44 | 36(81.82) | 8(18.18) |  |  | 37 | 35(94.59) | 2(5.41) |  |  |
| Allergen immunotherapy (after administration) | | No | 376 | 354(94.15) | 22(5.85) | p=1.000 |  | 281 | 275(97.86) | 6(2.14) | p=0.037 |  |
|  |  | Yes | 19 | 18(94.74) | 1(5.26) |  |  | 65 | 60(92.31) | 5(7.69) |  |  |
| Treatment for JC-pollinosis (baseline) | | No | 389 | 366(94.09) | 23(5.91) | p=1.000 |  | 339 | 328(96.76) | 11(3.24) | p=1.000 |  |
|  |  | Yes | 2 | 2(100.00) | 0(0.00) |  |  | 2 | 2(100.00) | 0(0.00) |  |  |
|  |  | Unknown | 4 | 4(100.00) | 0(0.00) |  |  | 5 | 5(100.00) | 0(0.00) |  |  |
| Treatments for JC-pollinosis^‡^ (after administration) | | No | 390 | 367(94.10) | 23(5.90) | p=1.000 |  | 345 | 334(96.81) | 11(3.19) | - |  |
|  |  | Yes | 2 | 2(100.00) | 0(0.00) |  |  | 0 | 0(-) | 0(-) |  |  |
|  |  | Unknown | 3 | 3(100.00) | 0(0.00) |  |  | 1 | 1(100.00) | 0(0.00) |  |  |
| Treatment for conditions other than JC-pollinosis^‡, \|\|^ (after administration) | | No | 392 | 369(94.13) | 23(5.87) | - |  | 345 | 334(96.81) | 11(3.19) | - |  |
|  |  | Yes | 0 | 0(-) | 0(-) |  |  | 0 | 0(-) | 0(-) |  |  |
|  |  | Unknown | 3 | 3(100.00) | 0(0.00) |  |  | 1 | 1(100.00) | 0(0.00) |  |  |
| † Fisher’s exact probability tests were performed for two-category analysis items, and χ^2^ tests were performed for three or more categories of analysis items | | | | | | | | | | | | |
| ‡ Aggregate data from both Season 1 and Season 2 | | | | | | | | | | | | |
| § Multiple answers possible | | | | | | | | | | | | |
| ¶ Concomitant drugs whose reason for use was “adverse event treatment” were excluded | | | | | | | | | | | | |
| \|\| Surgical treatments whose reason for use was “adverse event treatment” were excluded | | | | | | | | | | | | |
| JAU, Japanese allergy unit; JC, Japanese cedar; QoL, quality of life | | | | | | | | | | | | |
